# Acute care pathway assessed through performance indicators during the COVID-19 pandemic in OECD countries (2020–2021): a scoping review

**DOI:** 10.1186/s12873-024-00938-7

**Published:** 2024-01-26

**Authors:** Ana Sofia V. Carvalho, Bente Broekema, Óscar Brito Fernandes, Niek Klazinga, Dionne Kringos

**Affiliations:** 1 Amsterdam UMC Location University of Amsterdam, Public and Occupational Health, Meibergdreef 9, Amsterdam, the Netherlands; 2 Amsterdam Public Health Research Institute, Quality of Care, Amsterdam, the Netherlands; 3 Department of Pediatrics, Dijklander Hospital, Location Hoorn, Maelsonstraat 3, Hoorn, 1624 NP The Netherlands

**Keywords:** Quality of health care, Quality indicators, Quality indicators, Health care [MeSH], Continuity of patient care, Acute care, Emergency department, Emergency Service, Hospital [MeSH], COVID-19

## Abstract

**Background:**

The COVID-19 pandemic severely impacted care for non-COVID patients. Performance indicators to monitor acute care, timely reported and internationally accepted, lacked during the pandemic in OECD countries. This study aims to summarize the performance indicators available in the literature to monitor changes in the quality of acute care in OECD countries during the first year and a half of the pandemic (2020-July 2021) and to assess their trends.

**Methods:**

Scoping review. Search in Embase and MEDLINE (07-07-2022). Acute care performance indicators and indicators related to acute general surgery were collected and collated following a care pathway approach. Indicators assessing identical clinical measures were grouped under a common indicator title. The trends from each group of indicators were collated (increase/decrease/stable).

**Results:**

A total of 152 studies were included. 2354 indicators regarding general acute care and 301 indicators related to acute general surgery were included. Indicators focusing on pre-hospital services reported a decreasing trend in the volume of patients: from 225 indicators, 110 (49%) reported a decrease. An increasing trend in pre-hospital treatment times was reported by most of the indicators (*n* = 41;70%) and a decreasing trend in survival rates of out-of-hospital cardiac arrest (*n* = 61;75%). Concerning care provided in the emergency department, most of the indicators (*n* = 752;71%) showed a decreasing trend in admissions across all levels of urgency. Concerning the mortality rate after admission, most of the indicators (*n* = 23;53%) reported an increasing trend. The subset of indicators assessing acute general surgery showed a decreasing trend in the volume of patients (*n* = 50;49%), stability in clinical severity at admission (*n* = 36;53%), and in the volume of surgeries (*n* = 14;47%). Most of the indicators (*n* = 28;65%) reported no change in treatment approach and stable mortality rate (*n* = 11,69%).

**Conclusion:**

This review signals relevant disruptions across the acute care pathway. A subset of general surgery performance indicators showed stability in most of the phases of the care pathway. These results highlight the relevance of assessing this care pathway more regularly and systematically across different clinical entities to monitor disruptions and to improve the resilience of emergency services during a crisis.

**Supplementary Information:**

The online version contains supplementary material available at 10.1186/s12873-024-00938-7.

## Background

Since the World Health Organization (WHO) declared the COVID-19 outbreak a “Public Health Emergency of International Concern” [1] on the 30th of January, 2020, the COVID-19 pandemic has severely impacted health systems worldwide [2], alongside far-reaching economic and societal impact [3]. The surge of patients infected with SARS-CoV-2 led to staff deployment and reduced hospital capacity [4]. Furthermore, the containment measures enforced by national governments [5], patients’ hesitance to seek care [6, 7], and postponements of elective care contributed to disruptions in care pathways for non-COVID-19 patients, notably patients with noncommunicable diseases (NCDs) [8–10]. A care pathway or clinical pathway is a “structured multidisciplinary care plan” [11], which is used to translate evidence into local structures, to describe the steps of a treatment plan or care in a pathway or any “inventory of actions (i.e. the intervention has time-frames or criteria-based progression)” [11]. Furthermore, it intends to be a tool for care standardization for specific clinical problems in specific populations [11] and its ultimate aim is to improve quality of care [12]. Deleterious consequences of delayed care were reported among the Organization for Economic Co-operation and Development (OECD) member countries [9], such as increased mortality rates following hospital admissions with an acute cardiac event in several European countries in 2020 [8].

Emergency and trauma care are recognized by the WHO as part of Universal Health Coverage [13]. Emergency departments (EDs) are key to ensuring accessible, effective, and time-sensitive healthcare services, notably during crises [13, 14]. The WHO Emergency Care System Framework [15] provides a structured tool to appraise the essential functions of an emergency care system at the national level. During the pandemic, EDs worldwide faced the need to adapt quickly: services have reconfigured their structure to separate (potentially) infected from non-infected patients, build additional capacity, ensure physical distancing, and introduce new operational pathways, among others [16–20]. However, many OECD countries presented substantial data gaps to evaluate health services performance during this period [8], such as access and quality of ambulance and emergency care. To prepare emergency care systems in maintaining essential functions during crises, and ensure quality and equity of care [21], improvements in the regular monitoring of performance are paramount.

Healthcare performance measurement allows for regularly appraising health systems and informing policy-making [22]. Quality indicators can be defined as “quantitative measures that provide information about the effectiveness, safety and/or people-centeredness of care” [11]. Previous authors pursued efforts to identify indicators that could be used to monitor ED’s performance, from the literature and/or with consensus-based techniques [23–26]. Nonetheless, indicators that are fit for purpose and use [27], reported transparently, timely, internationally accepted, and grounded on robust health information systems lacked during the COVID-19 pandemic and are needed to guarantee the continuous monitoring of the quality of acute care provided and to tailor policy responses during a crisis [28, 29]. This scoping review intends to contribute to this knowledge, considering OECD member countries, which share a conceptual framework for performance measurement [30]. Therefore, we set out: 1) to summarize the performance indicators available in the literature, according to the phases of the acute care pathway, to monitor changes in the quality of acute care in OECD countries during the first year and a half of the COVID-19 pandemic (2020—July 2021) and 2) to assess their trends during this period.

## Methods

### Study design

We performed a scoping review following the methodological framework developed by Arksey and O’Malley [31] and Levac et al. [32]. Considering the heterogeneous methodologies of the studies assessing changes in the quality of care during the COVID-19 pandemic and the diversity of settings being studied [33], applying a scoping review methodology allows mapping the literature [32, 34, 35], specifically the performance indicators used to evaluate healthcare and the information they provide regarding changes in the quality of care [36]. Furthermore, it enables the synthesis and communication of emerging evidence [31, 34] and the identification of knowledge gaps [36]. This methodology has been described by the authors in two previous studies [37, 38], where different clinical areas were studied. The PRISMA checklist—extension for scoping reviews [39] was used for reporting (Additional file 1).

### Eligibility criteria

The following inclusion criteria were considered: 1) original scientific articles providing empirical data on the use of health services (including literature reviews); 2) studies displaying performance indicators related to NCDs during the COVID-19 pandemic; and 3) studies applying quantitative and/or qualitative methods. Studies were excluded when they did not provide empirical data on health services, namely: 1) editorials and commentaries; 2) prediction models; 3) clinical case reports; 4) health services organization or diseases management guidelines; 5) studies assessing the impact on healthcare workers, patients diagnosed with COVID-19, children, or pregnant women; 6) studies primarily performed in non-OECD countries; 7) conference abstracts. No limitations were set regarding language or year.

### Information sources & search strategy

The literature search was performed in Embase and MEDLINE databases. These two databases retrieve the most unique references in systematic reviews [40]. Relevant search terms were identified through pilot searches. The search strategy was elaborated by an experienced medical information specialist and refined following internal discussions among the research team. Search terms were grouped by key concepts: COVID-19, pandemic, noncommunicable disease, chronic disease, performance indicator, healthcare quality, healthcare utilization, and healthcare delivery. The information specialist conducted the search on 17–03-2021, which was updated on 14–12-2021 and 07–07-2022. The entire search strategy for Embase is available in Additional file 2. Duplicates were removed using EndNote reference management software.

### Study selection

Two reviewers (ASVC, OBF) screened titles and abstracts independently with Rayyan [41]. Disagreements were solved through discussion or consultation with a third reviewer (NK). Following the updates of the search strategy, one reviewer (ASVC) screened titles and abstracts. The records deemed relevant were exported to a spreadsheet. Only articles related to healthcare utilization of emergent and urgent care were considered for this study. Articles focusing exclusively on acute neurological, cardiac diseases, and trauma were excluded, considering that previous publications have extensively studied these subjects [37, 42–46]. Taking into consideration that most of the patients presenting with surgical diseases in the emergency department are evaluated by general surgeons, surgical diseases not evaluated in the general surgery setting were excluded. Full-text articles were independently screened for eligibility by two reviewers (ASVC, BB). Disagreements were solved through discussion and the reasons for exclusion were recorded at this stage. Articles in a language other than English, Dutch, French, Spanish, or Portuguese were translated to English with Google Translate, considering its accuracy [47].

### Data extraction and charting

A spreadsheet form was developed to collect data. First, we performed a pilot test with 10 studies, from which data were collected by one reviewer (BB) and revised by another (ASVC) (Additional file 3). Then, the spreadsheet form was consolidated through discussion. Subsequently, two reviewers (ASVC, BB) collected data independently, which were revised by one another. Extracted information from the studies comprised: generic and methodological aspects (e.g., title, country, study design), data regarding the indicators collected (e.g., indicator title as stated in the studies, numerator/denominator, inclusion/exclusion considerations), and the trend reported for every indicator (increase/decrease/stable). The trends regarding each indicator were collected as reported by the authors in each of the included studies. When the computation and/or the interpretation was not available, it was computed as: ((value during the COVID-19 pandemic) – (value before the COVID-19 pandemic)) / (value during the COVID-19 pandemic) * 100. The positive values were considered an increasing trend, the negative values were considered a decreasing trend, and values close to zero were considered stable.

### Data synthesis

Indicators collected from studies assessing emergent/urgent care without focusing on specific diseases were collated together. Indicators collected from studies explicitly related to general surgery in the ED were analyzed separately.

Indicators assessing identical clinical measures were grouped. Given the heterogeneity of indicator titles referring to equivalent measures, the authors attributed a common indicator title to each group of indicators assessing similar aspects of healthcare (eg, “volume of patients contacting emergency medical services”). The trends from each group of indicators were collated, and the percentage of indicators reporting each trend (increase/decrease/stable) was computed. For groups of indicators retrieved in low numbers (< 10 indicators), a trend was not computed. The groups of indicators and their combined trends were reported according to the phases of the acute care pathway: pre-hospital setting, access to the ED, diagnostic and treatment in the ED, and outcomes.

## Results

**Fig. 1:**
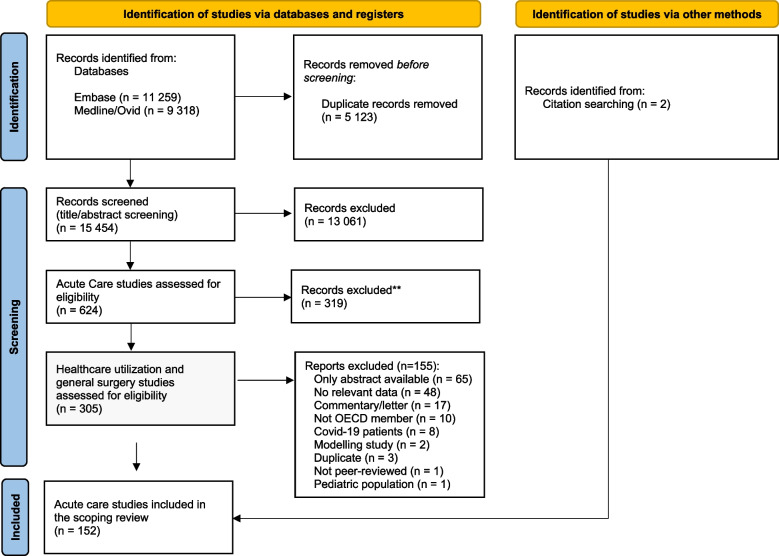
PRISMA flow diagram—literature search (17th March, 14th December 2021 and 07th July 2022). From: Page MJ, McKenzie JE, Bossuyt PM, Boutron I, Hoffmann TC, Mulrow CD, et al. The PRISMA 2020 statement: an updated guideline for reporting systematic reviews. BMJ 2021;372:n71. https://doi.org/10.1136/bmj.n71

Database searches retrieved 15454 records. Following screening of titles and abstracts, 2393 studies met the inclusion criteria. From those, 624 acute care studies were assessed for eligibility, from which 319 were excluded, considering the clinical areas excluded for this review. A total of 305 acute care studies focusing on healthcare utilization and general surgery were considered for full-text screening. Two records were identified via hand-searching. Following the full-text screening, 152 were included and 155 studies were excluded (reasons for exclusion detailed in Fig. 1).

### Studies characteristics

A total of 152 studies were included (Additional file 4), conveying information from 27 countries. The most frequent study design was the retrospective cohort (*n* = 141; 92.8%), followed by 5 prospective cohort studies (3.3%), 4 systematic reviews (2.6%), and 2 surveys (1.3%). Administrative data (*n* = 92; 56.8%) and clinical data (*n* = 49; 30.2%) were the most frequent data sources, followed by registries (*n* = 8), claims (*n* = 4), population-level data (*n* = 3), and surveys (*n* = 2). The most frequent countries reported on, excluding the literature reviews, were the United States of America (USA) (*n* = 39; 26.4%), Italy (*n* = 21; 14.2%), Canada (*n* = 12; 8.1%) and the United Kingdom (UK) (*n* = 10; 6.8%) (Table 1). The magnitude of the indicators in the year 2020, in each study, were compared to their magnitude in 2019 (*n* = 240; 36.9%), to the period immediately before the beginning of the pandemic (*n* = 162; 24.9%), or to the average in previous years, ranging from 2018 to 2019 (*n* = 111; 17.1%). Some studies compared the first half of the year 2021 to 2019 (*n* = 103; 15.8%) or the first half of the year 2021 to the previous years (*n* = 34; 5.2%).

**Table 1:** OECD countries included in the analysis, excluding literature reviews (*n* = 148 studies)

Country	Number of studies included	
	**n**	**%**
United States of America	39	26.4%
Italy	21	14.2%
Canada	12	8.1%
United Kingdom	10	6.8%
Turkey	9	6.1%
Germany	8	5.4%
United Kingdom	5	3.4%
Israel	5	3.4%
Australia	5	3.4%
Japan	4	2.7%
France	4	2.7%
Ireland	4	2.7%
Spain	3	2.0%
Finland	3	2.0%
Switzerland	3	2.0%
Greece	2	1.4%
Scotland	1	0.7%
Austria	1	0.7%
Slovenia	1	0.7%
Croatia	1	0.7%
Korea	1	0.7%
Netherlands	1	0.7%
Belgium	1	0.7%
New Zealand	1	0.7%
Hungary	1	0.7%
Norway	1	0.7%
Portugal	1	0.7%

### Acute care indicators

A total of 2953 indicators related to acute care were collected: 2354 indicators from 124 studies focusing on general emergent/urgent healthcare and 301 indicators from 28 studies related to acute general surgery (Table 2).

**Table 2:** Groups of performance indicators across the acute care pathway (*n* = 2953 indicators)

	Number of indicators^a^		Number of studies
	n	%	n
***General emergent and urgent healthcare***			
**1. Pre-hospital services**		**13.6%**	
1.1. Volume of patients contacting Emergency Medical Services	225		20
1.2. Volume of bystander cardiopulmonary resuscitation	26		10
1.3. Proportion of patients with out-of-hospital cardiac arrest witnessed by bystanders	12		9
1.4. Pre-hospital treatment times: from call to care provided	59		12
1.5. Survival rate of patients with out-of-hospital cardiac arrest	81		14
**2. Admission to the Emergency Department**		**46.2%**	
2.1. Volume of Emergency Department visits	1066		87
2.2. Arrival mode to the Emergency Department	78		22
2.3. Level of urgency by triage system	186		34
2.4. Clinical severity at admission	26		8
2.5. Duration of symptoms prior to presentation^b^	9		5
**3. Diagnosis**		**5.5%**	
3.1. Volume of diagnostic procedures	131		16
3.2. Rate of positive findings after diagnostic testing	32		4
**4. Treatment**		**4.9%**	
4.1. Volume of therapeutic interventions	106		18
4.2 Operational times	17		10
4.3 Length of stay (Emergency Department / in-hospital)	22		16
**5. Outcomes**		**9.3%**	
5.1 Disposition after visit to the Emergency Department	228		49
5.2 Unscheduled returns / re-admissions^b^	3		3
5.3 Scheduled follow-up visit after Emergency Department visit^b^	2		2
5.2 Mortality rate after Emergency Department admission	43		23
***General surgery in the Emergency Derpartment***			
**1. Admission to the Emergency Department**		**5.7%**	
1.1. Volume of Emergency Department visits	101		17
1.2. Clinical severity at admission	68		19
**2. Diagnosis**		**0.3%**	
2.1. Volume of diagnostic procedures	8		4
**3. Treatment**		**3.3%**	
3.1. Volume of emergency surgeries	30		10
3.2. Change in treatment approach	43		19
3.3. Length of hospital stay	19		20
**4. Outcomes**		**0.9%**	
4.1 Post-operative complications	10		8
4.2 Mortality rate	16		12

^a^A total of 298 indicators were not included in the analysis since they were too specific to be grouped into categories

^b^Indicator retrieved in low numbers therefore it is not mentioned in the text and a trend was not computed

## Indicators related to general emergent and urgent care

**Fig. 2:**
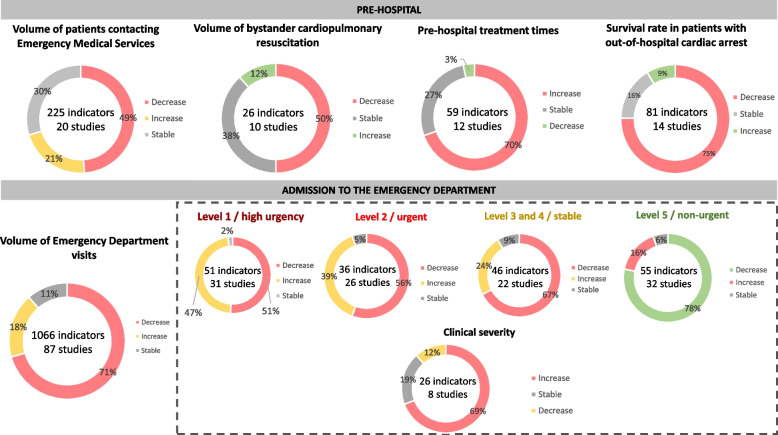
Group of general acute healthcare indicators and their respective trends: OECD countries (2020 – July 2021)

Indicators and their trends were collated according to the acute care pathway (pre-hospital services, admission to the emergency department, diagnosis, treatment, and outcomes), which are outlined as follows (Fig. 2).

### Pre-hospital services

#### Volume of patients contacting emergency medical services

A total of 225 indicators from 20 studies [48–67] assessed the volume of patients contacting Emergency Medical Services (EMS). From those, most of the indicators (*n* = 110; 49%) showed a decreasing trend. These data came predominantly from Australia (*n* = 73 indicators; 33%) and Canada (*n* = 55 indicators; 24%).

One systematic review [68] reporting data from Australia, France, Italy, Spain, and the USA showed an increase of 120% in the number of out-of-hospital cardiac arrest (OHCA) events when comparing the year 2020 with a previous period.

#### Volume of bystander cardiopulmonary resuscitation

Twenty-six indicators from 10 studies [48, 56, 59, 60, 65, 66, 69–72] evaluated the volume of bystanders providing first aid in collapsed patients out-of-hospital. These indicators revealed, in most cases, a decreasing trend (*n* = 13;50%). Most data were from the USA (*n* = 9;35%) and France (*n* = 6;23%). Similarly, one systematic review [68] documented a decrease in bystander cardiopulmonary resuscitation (BCPR) rates during the pandemic, although not statistically significant. Two meta-analyses [73, 74] revealed no statistical difference in BCPR rates.

#### Proportion of patients with out-of-hospital cardiac arrest witnessed by bystanders

Twelve indicators from 9 studies [48, 56, 59, 60, 65, 66, 69–71] reported on the proportion of patients with OHCA witnessed by bystanders, most of those (7;59%) showed stability in this proportion.

#### Pre-hospital treatment times

The analysis of the treatment times in the pre-hospital setting revealed 59 indicators from 12 studies [48, 50, 52, 55, 57, 59, 63, 65, 69–72], predominantly from Australia (*n* = 14; 27%) and Finland (*n* = 12;20%). Most indicators (*n* = 41;70%) signaled an increasing trend. The treatment times assessed included: “response time: start of an emergency call to the arrival of the first ambulance at the scene” [63], “activation time: from the time someone reports to EMS to the time EMS departure” [72], “on-scene time: from the time arrival at scene to the time of departure for hospital” [72], “transport time: departure from the scene to arrival at the hospital” [63], among others. Similarly, the systematic reviews that assessed pre-hospital treatment times [68, 73, 75] documented significantly increased times from OHCA to ambulance arrival during the pandemic.

#### Survival rate of patients with out-of-hospital cardiac arrest

Concerning the survival rate of patients with out-of-hospital cardiac arrest, 81 indicators from 14 studies [48, 51, 52, 55–57, 59, 60, 64–66, 70, 72, 76] were identified. These indicators included the following sub-categories of indicators: “on-scene death rate”, “rate of pre-hospital return of spontaneous circulation (ROSC)”, “survival rate to hospital admission”, and “survival rate to hospital discharge”. Most indicators (*n* = 61;75%) signaled a decreasing trend in survival rates. Data came predominately from Spain (*n* = 44;62%) and the USA (*n* = 20;26%). Similarly, three of the systematic reviews documented a significant increase in the mortality rate following OHCA during the pandemic [68], lower rates of pre-hospital ROSC [74, 75], decreased survival rates to hospital admission [74] and to hospital discharge [74, 75].

### Admission to the emergency department

#### Volume of emergency department visits

A total of 1066 indicators from 87 studies [50, 52, 61, 67, 76–158] provided information on the volume of patients admitted to the Emergency Department. From these, 752 (71%) indicators displayed a decreasing trend. Most of the data came from the USA (*n* = 309;29%), Italy (*n* = 163;15%), and the UK (*n* = 122;11%).

#### Arrival mode to the emergency department

Three indicator categories were identified regarding the arrival mode of patients to the ED. A total of 16 indicators from 9 studies [67, 77, 81, 86, 93, 110, 124, 133, 137] assessed the *volume of patients arriving with their own transport*, from which 15 (94%) indicators showed a decreasing trend. Twenty-two indicators from 12 studies [52, 67, 77, 81, 86, 91, 110, 112, 124, 133, 137, 159] evaluated the *volume of patients reaching the ED with the Emergency Medical Services*. Most indicators (13;57%) signaled a decreasing trend. One study from Germany [110] displayed 40 indicators evaluating the number of ED admissions with a *referral by a doctor* related to different diseases; of those, 29 (72%) indicators showed a decreasing trend.

#### Level of urgency by a triage system

A total of 51 indicators from 31 studies [52, 62, 77, 80–82, 86, 88–91, 97, 98, 102, 103, 105, 108, 113, 116, 119, 123, 124, 126, 130–132, 134, 137, 142, 144, 158] were identified regarding patients presenting with *level 1/highly urgent conditions* to ED, mainly from the USA (*n* = 21;41%) and Italy (*n* = 13;25%). Of these indicators, 51% (*n* = 26) signaled a decreasing trend. Of 36 indicators from 26 studies [52, 62, 77, 80–82, 86, 88, 91, 97, 98, 102, 103, 105, 108, 113, 123, 124, 126, 130, 134, 137, 142, 151, 157, 158] reporting on the volume of patients presenting with *level 2/urgent conditions* to ED, 56% (*n* = 20) reported a decreasing trend. These data came predominantly from the USA (*n* = 14;39%) and Italy (*n* = 6;16%). Concerning the *stable conditions/levels 3 and 4*, 46 indicators from 22 studies [52, 62, 77, 81, 86, 91, 97, 98, 102, 108, 113, 116, 119, 123, 126, 130, 131, 134, 137, 142, 144, 158] provided information on indicators’ trends from 7 countries. Most of these indicators reported a decreasing trend (*n* = 31;67%). Data came mainly from the USA (*n* = 23;50%). Concerning *non-urgent/level 5* conditions, a total of 55 indicators from 32 studies [52, 62, 77, 80–82, 86, 88–91, 97, 98, 102, 108, 113, 116, 119, 123, 124, 126, 130–132, 134, 137, 142, 144, 151, 156–158] were identified. Of these, 78% (*n* = 43) reported a decreasing trend in the volume of these patients. Data were reported predominantly from the USA (*n* = 20;36%) and Italy (*n* = 15;27%).

#### Clinical severity at admission

Regarding patients’ clinical severity at admission, 26 indicators from 8 studies [83, 103, 120, 136, 143, 159–161] were identified. Most indicators reported an increasing trend (*n* = 18;69%). Data were reported mainly from the UK (*n* = 8.31%) and Switzerland (*n* = 5;19%). The indicators retrieved were related to the overall severity of medical admissions [120], clinical severity of patients diagnosed with urolithiasis [136], diverticulitis [160], pyelonephritis [83], pulmonary embolism [103], chronic obstructive pulmonary disease [143], and appendicitis [159].

### Diagnosis

#### Volume of diagnostic procedures

Information on the volume of diagnostic procedures in the ED was retrieved from 131 indicators from 16 studies [82, 86, 92, 95, 103, 113, 125, 145, 160–167] reporting on 8 countries. They displayed information regarding laboratory testing [162, 168], ultrasound imaging [86, 166], radiological examinations [86, 92, 103, 125, 145, 160–167], nuclear imaging [86, 166], and biopsies [95]. Most of the indicators reported on computed topographies (CT) (*n* = 64;49%) and laboratory testing (*n* = 16;6%). The predominant trend was a decrease in the volume of diagnostic procedures (*n* = 85,65%) (trends for each diagnostic procedure are detailed in Additional file 5). Data came predominantly from the USA (*n* = 74 indicators;56%) and Italy (*n* = 19;14%).

#### Rate of positive findings after diagnostic testing

A total of 32 indicators from 4 studies reporting on 4 countries were retrieved regarding the rate of positive diagnostic findings, namely positive blood cultures [162] and positive CT findings [92, 161, 162, 164]. Of these, 9 (58%) indicators displayed an increasing trend. Most indicators were from the USA (*n* = 18; 55%) and Ireland (*n* = 7; 21%).

### Treatment

#### Volume of therapeutic interventions

A total of 106 indicators from 18 studies [67, 82, 83, 88, 92, 93, 95, 98, 99, 108, 136, 148, 151, 155, 159, 161, 169, 170] and 11 countries reported on therapeutic interventions in the ED. Most indicators signaled a decreasing trend (*n* = 54; 51%). The therapeutic interventions assessed were invasive procedures (e.g., endoscopy, endovascular intervention, endoscopic retrograde cholangiopancreatography, paracentesis, thoracentesis), acute surgical interventions, and other procedures, such as bedside procedures.

#### Operational times

Seventeen indicators from 10 studies and 7 countries reported on the average operational times for emergent and urgent care, such as average waiting time for triage [123, 167], from triage to first medical assistance [52, 123, 126, 130], from consultation to treatment [52, 90, 148, 161, 162], and for hospital ward admission [131]. Of these, 9 (53%) indicators signaled a decreasing trend.

#### Length of stay

A total of 14 indicators from 11 studies [52, 86, 91, 98, 114, 120, 126, 130, 145, 151, 157] reported on the *length of stay (LOS) in the ED*, from which 11 (79%) indicators displayed a decreasing trend. Concerning the in-hospital LOS after ED admission, 8 indicators from 5 studies [127, 130, 139, 143, 159] were retrieved, from which 62% (*n* = 5) reported a decreasing trend.

### Outcomes

#### Disposition after visit to the Emergency Department

Concerning the *volume of patients discharged home*, a total of 26 indicators from 15 studies [52, 82, 86, 98, 102, 112, 113, 116, 117, 120, 130, 136, 151, 157, 158] and 5 countries were retrieved. Most indicators (*n* = 15;58%) reported a decreasing trend. Regarding the *volume of hospital admission after ED presentation* (including admission to intensive care unit), from the 191 indicators from 46 studies [52, 67, 77, 78, 81, 83, 85–88, 90–94, 96, 98, 100, 102, 103, 107, 108, 112–117, 120–123, 127, 128, 130, 131, 134, 137, 144, 145, 147, 149, 151, 152, 157, 158] collected, 52% (*n* = 100) signaled a decreasing trend, and 39% (*n* = 74) showed an increasing trend. Most of the indicators were from the USA (*n* = 43;23%), Italy (*n* = 35;18%), and Croatia (*n* = 23;12%). Eleven indicators from 7 studies [77, 98, 113, 130, 151, 157, 171] and 3 countries reported on the *volume of patients leaving the ED without completing treatment*, including patients that left the ED without being seen, completing treatment, and against medical advice; of these, 9 (82%) indicators signaled a decreasing trend.

#### Mortality rate

A total of 43 indicators from 23 studies [50, 52, 67, 76, 85, 91, 92, 96, 98, 107, 113, 120, 123, 127, 130, 133, 137, 139, 143, 149, 153, 157, 162] reported on the mortality rate after an ED visit. Most of the indicators signaled an increasing trend (*n* = 23;53%). Data came predominantly from Canada (*n* = 9;21%), Turkey (*n* = 6;14%), and Norway (*n* = 5;11%).

Trend analysis by country and clinical entity did not show recognizable tendencies in any of the indicators retrieved (Additional file 5). The trends of the indicator categories related to general acute care comparing the COVID-19 period to a previous period are displayed in Fig. 2.

## Indicators related to acute general surgery care

**Fig. 3:**
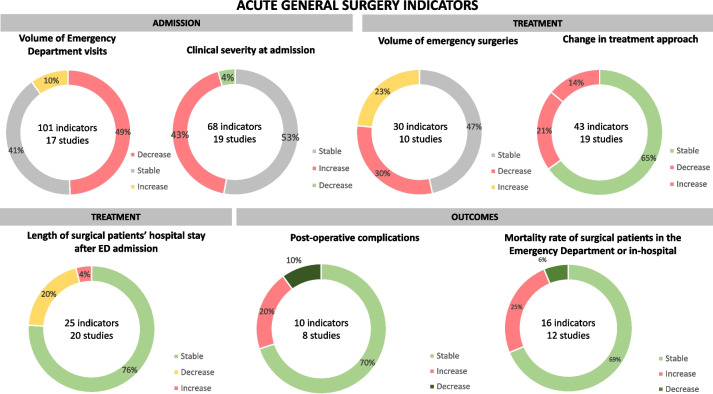
Groups of acute general surgery indicators and their respective trends: OECD countries (2020 – July 2021)

Indicators on acute general surgery care were collected and grouped following the care pathway (admission, diagnosis, treatment, and outcomes), and their respective trends are summarized in Fig. 3.

### Admission

#### Volume of emergency department visits

A total of 101 indicators from 17 studies [172–188] and 10 countries reported on the volume of ED visits related to acute general surgery. Of those, almost half of the indicators (*n* = 50;49%) signaled a decreasing trend. Most of the data were from Canada (*n* = 19;18%), the UK (*n* = 14;14%), and New Zealand (*n* = 12; 12%).

#### Clinical severity at admission

Indicators assessing patients’ clinical severity included clinical, laboratory and imaging features at admission, severity grading according to diseases-specific scores, operative findings, and the American Society of Anesthesiologists (ASA) score. Sixty-eight indicators were retrieved from 19 studies; of those, 36 (53%) indicators showed a stable clinical severity and 29 (43%) indicators signaled increased severity. The countries most represented were the USA (*n* = 19;28%), Israel (*n* = 15; 22%), and the UK (*n* = 11;16%).

### Diagnosis

Eight indicators from four studies evaluated the diagnosis phases of the pathway, which did not provide sufficient information regarding trends.

### Treatment

#### Volume of emergency surgeries

Data related to the volume of acute surgeries was retrieved from 30 indicators and 10 studies [174, 176, 185, 186, 188–193], from 8 countries. Most of the indicators (*n* = 14;47%) signaled stability in the volume of acute surgeries. Data came predominantly from Italy (*n* = 16;54%).

#### Change in treatment approach

A total of 43 indicators from 19 studies [173–177, 179, 180, 182, 183, 186, 188–191, 194–198] were retrieved. These indicators evaluated the treatment strategy in the acute surgical setting related to diseases such as appendicitis, acute cholecystitis, and bowel obstruction. Most of the indicators (*n* = 28;65%) signaled no change in the treatment approach. Data were predominantly from the UK (*n* = 12;28%) and Germany (*n* = 9;21%).

#### Length of hospital stay

Concerning the LOS in-hospital (including in the intensive care unit), 25 indicators from 20 [175–181, 183, 184, 186, 189–191, 193–199] studies were collected. Most of the indicators showed stability of the LOS (*n* = 19;76%). Data came mostly from the UK (*n* = 7;28%), USA (*n* = 4;16%), and Ireland (*n* = 4; 16%).

### Outcomes

#### Post-operative complications

Ten indicators from 8 studies [174, 175, 179, 182, 186, 194–196] reported on post-operative complications. Most of these indicators (*n* = 7;70%) signaled stability in this outcome.

#### Mortality rate

Concerning the mortality rate, 16 indicators from 12 studies [174, 176–178, 182, 183, 186, 187, 189, 190, 195, 198] were collected. Of these, 69% (*n* = 11) signaled stability.

## Discussion

In this scoping review, we aimed to summarize the performance indicators available in the literature to monitor changes in the quality of acute care in OECD countries and to assess their trends for the first year and a half of the COVID-19 pandemic (2020-July 2021). A total of 2953 indicators were collected from 152 studies reporting on 27 countries. Concerning access to acute care, indicators signaled a decreasing trend in the volume of patients, as well as increased clinical severity at presentation, and treatment delays. Trends regarding acute diagnostic and treatment procedures showed decreases in volume, as well as worsened outcomes. Similarly, the subset of indicators focusing on acute general surgery signaled a decrease in the volume of patients and increased clinical severity. Conversely, our results showed stable trends in acute general surgery regarding the volume of surgeries, length of hospital stay, treatment approaches, and outcomes.

Looking in detail at the stages of the acute care pathway, starting from the pre-hospital setting, our results showed a decrease in the volume of patients contacting EMS, which is in line with findings of other studies reporting reductions in access to primary care [8] and to hospital care by patients with acute myocardial infarction (AMI) [8], cancer [200], and stroke [201]. Our findings support that patients delayed care considering the increased time from symptom until calling the EMS, as reported in previous studies [202]. The decision to delay care might be related to fear of overwhelming the health system [7, 142] or getting infected with SARS-CoV2 [203]. This fear could also explain the decreasing trend in bystanders providing first aid that our results show. The possibility of reductions in the incidence of some diagnoses is a hypothesis to consider. However, it is difficult to quantify the exact number of “missed” diagnoses during the COVID-19 pandemic, as these are affected by several factors, such as changes in the mortality rates, lifestyle, or migration in each country. A study from the UK, conducting an analysis between March 2020 and December 2021, found a persistent decrease in the incidence rate of some respiratory diseases (such as asthma and chronic obstructive pulmonary disease), strokes, and heart diseases (such as coronary heart disease and atrial fibrillation), when compared to pre-pandemic levels [204]. Concerning stroke, the avoidance in care seeking is generally hypothesized as one of the justifications rather than the hypothesis of a real decrease in the incidence of stroke [205, 206]. Regarding the respiratory conditions, the social distance measures may have led to a reduced number of acute exacerbations of these diseases. Nonetheless, the restrictions in access to diagnostic testing (spirometry) could also have contributed to this decrease [204]. The impact of the pandemic in the outcomes of patients with cardiac diseases remains unclear. Factors such as the misclassification of cardiovascular deaths as COVID-19 deaths, the lack of population data and the lack of regular monitoring of the quality of cardiovascular care and outcomes makes it difficult to formulate accurate conclusions [207].

Despite the reduced demand, our results signaled an increasing trend in pre-hospital times, suggesting the timeliness of acute care was compromised. Delays triggered by patients, as signaled by our results, and insufficient timely response have most likely contributed to the worsening of survival rate trends in patients with OHCA that we report. Some authors have also reported increased pre-hospital times and worsened outcomes in patients with acute stroke [202] and AMI [208].

Regarding access to the ED, our results also signal substantial disruptions. We found a decreasing trend in the volume of patients regardless of triage classification or mean of arrival. This decrease was neither condition- nor country-specific, suggesting that fear and delay were relevant for patients with different clinical conditions and across different countries. Our results also signal a trend toward increased clinical severity at admission to the ED, as previous studies reported for specific diseases such as acute stroke [203]. These delays in access to pre-hospital care and to the ED highlight the need to improve communication strategies directed to patients with symptoms related to time-sensitive and life-threatening conditions during crises. Care provided at the ED should primarily focus on the most urgent patients, therefore the decreasing trend in access by non-urgent patients could provide a window of opportunity to avoid unjustified use of the ED, emphasizing the need to strengthen and integrate primary care support.

The reduced demand for access to acute care settings resulted in decreasing trends in acute diagnosis and treatment procedures. Noteworthy, our results also showed higher rates of positive findings after diagnostic testing, which is in line with the more severe clinical condition displayed in our results.

Our results feature a small sample of indicators concerning the timeliness of care in the ED that signal a decreasing trend in operational times and in LOS in the ED. Although this small sample of indicators was insufficient to compare trends among different periods, previous studies reported variations in the LOS during 2020 [209, 210] and for different clinical severities [211].

Regarding the outcomes after ED admission, we report a decreasing trend in the volume of patients discharged home and in the volume of hospital admissions, which are most likely related to the reduced demand. Additionally, our results signal a trend towards increased mortality rates after admission to the ED. This adverse outcome could be a consequence of delays in seeking care and increased pre-hospital treatment times. Worse clinical outcomes were previously reported for specific acute diseases during the pandemic, such as acute stroke [202] and AMI in several European countries [8, 212].

The analysis of the subset of indicators assessing acute general surgery care did not show similar disruptions. We found a decreasing trend in the volume of patients at the ED, however our results display no change in treatment approaches, length of stay, and outcomes. The small sample of indicators needs to be taken into consideration to interpret these findings. Notwithstanding, these results suggest that the quality of care provided to these patients has not substantially changed during the pandemic. The difference of these results compared to the general acute healthcare justify the need to monitor the pathways of acute diseases separately to identify and act upon changes in the quality of care regarding different clinical entities in a timely manner.

The disruptions signaled by these results highlight that the inclusion of this performance information as part of the continuous monitoring and more systematic assessment of emergency care systems is pivotal to tailoring policy responses timely and to building more resilient health systems. Performance intelligence, defined as “the structured approach to acting on health policies, using knowledge and information generated by the application of scientific methods to comparable healthcare data to systematically measure indicators of health systems performance” [28], can be a valuable tool to prepare for future crises [28]. Investment in data infrastructure is an essential component to enable performance intelligence [28] and to improve healthcare systems resilience [213]: the “ability of systems to prepare for, absorb, recover from and adapt to major shocks” [213].

Substantial advancements to health information systems were triggered by the COVID-19 pandemic, such as improved data linkage between data sets in the public sector, improved timeliness of national datasets, and adoption of digital tools [29]. Nonetheless, relevant hindrances still need to be addressed, namely related to regulatory barriers, data interoperability, quality and linkage, as well as the need for a skilled workforce in health information [29]. Good practices to inform further improvements are, for instance, Korea’s Emergency Medicine Monitoring System, a nationwide information network constructed in 2013 that links public sources to calculate key indicators at the entire acute care pathway, providing real-time information [168, 214, 215]. The Israeli National Hospital Discharge Register [216] also assesses performance indicators related to stroke and AMI regularly.

Our results expose only a few of indicators reflecting the impact on outcomes and experiences of patients or patient safety culture in hospitals, both recognized as relevant for international benchmarking and healthcare quality improvement [8, 217]. Future research is needed to provide a deeper analysis of the impact on specific acute clinical entities in a comparable way across countries. Moreover, stratified analyses of these indicators by patients’ sociodemographic characteristics will be useful to enhance the understanding of the trends we report and assess possible inequalities. Future studies to develop performance intelligence that is actionable to decision-making will be crucial in the years to come.

### Strengths and limitations

Previous studies have investigated the impact of the COVID-19 pandemic in the acute care setting on specific countries [24, 209, 211, 218], phases of the pathway [142, 211], or diseases [203, 212, 219]. This scoping review provides a comprehensive overview of the changes reported on the quality of acute care during the first year and a half of the COVID-19 pandemic, covering OECD countries, multiple diseases, and following a care pathway approach. In addition, this review provides a summary of performance indicators from pre-hospital to in-hospital outcomes, which could be useful tools, if further developed and systematically collected, to inform policymaking. Some national and international reports [9, 220] proposed standards for acute care, where some of the performance indicators we retrieved were proposed to drive care improvements.

This study has some limitations. First, the heterogeneity of study designs, sample sizes, and indicators definitions did not allow to perform a meta-analysis to quantify the impact of the COVID-19 pandemic on the indicator categories identified, or to assess inequalities related to gender or socio-economic status. Secondly, despite the inclusion of all OECD countries, the heterogeneity in the number of studies per country comprised a skewed sample. Therefore, it needs to be considered that the professionals experiencing the most severe disruptions were likely the ones that were more inclined to report it.

## Conclusion

This study signals relevant disruptions across the acute care pathway during the first year and a half of the COVID-19 pandemic, such as decreasing trends in the volume of patients, in the volume of diagnostic and treatment procedures, increased clinical severity at presentation, treatment delays, and worsened outcomes. A subset of general surgery indicators showed stability in most of the phases of the care pathway. This evidence underscores the relevance of monitoring the acute care pathway more regularly and systematically to assess the care processes and outcomes of these patients. This would allow to monitor care disruptions more efficiently. Furthermore, our results could contribute to strengthening health information infrastructures worldwide, which is an urgent step to monitor structure, process, and outcome information regarding patients with time-sensitive and life-threatening conditions. This is of paramount importance to improve the resilience of health systems during crises.

### Supplementary Information

**Additional file 1.** Preferred Reporting Items for Systematic reviews and Meta-Analyses extension for Scoping Reviews (PRISMA-ScR) Checklist.

**Additional file 2.** Search strategy.

**Additional file 3.** Data Extraction Form.

**Additional file 4.** List and characteristics of the studies included, from which indicators were extracted and collated.

**Additional file 5.** Analysis of specific indicator trends by country, clinical entity, and/or diagnostic procedures.

## Data Availability

The datasets generated and analysed during the current study are freely and openly available in the Zenodo.org repository, at https://doi.org/10.5281/zenodo.7726234 [221].

## Abbreviations

AMI: Acute myocardial infarction
ASA: American Society of Anesthesiologists
ASVC: Ana Sofia V. Carvalho
BB: Bente Broekema
BCPR: Bystander cardiopulmonary resuscitation
CT: Computed topographies
EMS: Emergency Medical Services
LOS: Length of stay
NCDs: Non-communicable diseases
NK: Niek Klazinga
OBF: Óscar Brito Fernandes
OECD: Organisation for Economic Co-operation and Development
OHCA: Out-of-hospital cardiac arrest
ROSC: Return of spontaneous circulation
UK: United Kingdom
USA: United States of America
