# Supplementary material for: Acute care pathway assessed through performance indicators during the COVID-19 pandemic in OECD countries (2020–2021): a scoping review
Source: BMC Emerg Med. 2024 Jan 26;24:19. doi: 10.1186/s12873-024-00938-7 (PMC10811879; doi:10.1186/s12873-024-00938-7)
Supplement: Supplementary file 2 — Additional file 2. Search strategy. [file 12873_2024_938_MOESM2_ESM.docx]

**S2 Doc – Search strategy**

**Embase.com**

(pandemic/mj/exp OR 'natural disaster'/mj/exp OR disaster/mj/exp OR hurricane/mj/exp OR earthquake/mj/de OR 'coronavirus disease 2019'/mj/de OR 'Severe acute respiratory syndrome coronavirus 2'/mj/de OR (pandemic* OR disaster* OR hurricane* OR flood OR flooding OR earthquake* OR coronavirus-disease-2019 OR covid19 OR covid-19 OR sars-cov-2 OR 2019-novel-coronavirus OR 2019-ncov OR lockdown OR lock-down OR typhoon* OR (natural NEAR/3 hazard*)):ti) AND ('non communicable disease'/de OR neoplasm/exp OR 'malignant neoplasm'/de OR 'cancer patient'/exp OR 'heart disease'/exp OR cardiology/de OR 'neurologic disease'/de OR 'cancer screening'/de OR 'cancer surgery'/de OR (('primary medical care'/exp OR 'general practitioner'/de OR 'general practice'/de OR 'family medicine'/de OR 'emergency ward'/exp OR 'emergency care'/de) AND ('health care utilization'/exp OR consultation/de)) OR 'diabetes mellitus'/exp OR 'rheumatic disease'/exp OR 'mental health service'/de OR 'cardiovascular disease'/exp OR 'cerebrovascular disease'/exp OR 'chronic kidney failure'/exp OR 'obstructive airway disease'/exp OR 'chronic respiratory tract disease'/de OR 'cerebrovascular accident'/exp OR hypertension/exp OR 'chronic disease'/de OR 'neurologic disease'/exp OR (((non-communicab* OR noncommunicab*) NEAR/3 disease) OR cancer OR ((heart OR cardiovascul* OR cerebrovascul*) NEAR/3 (disease* OR patient* OR emergenc*)) OR cardiolog* OR neurolog* OR oncolog* OR ((((emergency OR acute) NEAR/3 (ward* OR care OR department*)) OR (general NEXT/1 (practi*)) OR ((primary OR family) NEXT/2 (healthcare* OR care OR doctor*))) AND (utilization* OR utilisation* OR delay* OR time OR visit* OR consultation* OR barrier* OR access* OR challenge* OR vulnerab* OR attendan* OR hesita*)) OR diabet* OR rheumat* OR (mental NEAR/3 (health-care* OR health-service* OR healthcare*)) OR (chronic NEAR/3 (kidney OR renal OR respirator* OR lung* OR pulmonar*) NEAR/3 (failure OR disease*)) OR asthma* OR bronchitis OR ((lung* OR pulmonar*) NEAR/3 (emphysem*)) OR ((cerebrovascul* OR cerebro-vascul*) NEAR/3 accident*) OR stroke OR hypertens* OR chronic-disease* OR (surg* NEAR/3 emergenc*)):ab,ti) AND ('disease exacerbation'/mj/de OR 'recurrent disease'/mj/de OR 'avoidance behavior'/mj/de OR 'recurrence risk'/mj/de OR 'undiagnosed disease'/mj/de OR 'diagnostic error'/mj/de OR 'diagnostic delay'/mj/de OR 'delayed diagnosis'/mj/de OR 'therapy delay'/mj/de OR 'health care utilization'/mj/de OR mortality/mj/exp OR morbidity/mj/exp OR 'health care quality'/mj/exp OR screening/mj OR 'mass screening'/exp/mj OR 'screening test'/mj OR 'palliative therapy'/exp/mj OR 'health care delivery'/mj OR 'health care access'/exp/mj OR 'surgical volume'/mj OR rehabilitation/exp/mj OR 'performance indicator'/de OR (impact* OR influence* OR affect* OR effect* OR exacerbate* OR (Disease NEAR/3 progression*) OR recur* OR avoid* OR postpon* OR post-pon* OR implication* OR ((diagnos* OR therap* OR treatment* OR hospitali* OR services OR visit*) NEAR/3 (error* OR delay* OR fewer* OR drop OR decline* OR continuit* OR decrease* OR increase* OR reduc*)) OR ((health-care OR healthcare) NEAR/3 (utilizat* OR utilisat* OR use)) OR mortalit* OR morbidit* OR ((health-care OR healthcare) NEAR/3 (quality)) OR screening OR (service* NEAR/3 ( disrupt* OR continu*)) OR palliat* OR unmet OR rehabilitation*):ti OR (((performance* OR outcome*) NEAR/3 (indicator*)) OR ((health-care OR healthcare) NEAR/3 (access* OR deliver* OR output*)) OR ((surgical OR surgeries OR procedures) NEAR/3 (volum* OR number*))):ab,ti) NOT (model/exp/mj OR (model*):ti) NOT ([conference abstract]/lim AND [2000-2019]/py) NOT ([animals]/lim NOT [humans]/lim) NOT ('health care personnel'/exp/mj OR pregnancy/exp/mj OR 'pregnant woman'/mj OR (((COVID-19 OR COVID19 OR coronavirus* OR corona-virus* OR SARS-CoV-2) NEAR/3 (outcome* OR case* OR patient* OR pneumoni* OR progression* OR prognos* OR mortalit* OR fatal* OR sever* OR vaccine*)) OR pregnan* OR ((healthcare OR health-care OR medical) NEAR/3 (personnel* OR staff OR worker* OR workforce* OR work-force*)) OR doctor* OR nurse* OR physician*):ti OR ((COVID-19 OR COVID19 OR coronavirus* OR corona-virus* OR SARS-CoV-2) NEAR/3 (mortalit* OR outcome* OR fatal*)):ab) NOT ((child/exp NOT adult/exp) OR (pediatr* OR paediatr* OR child* OR infan* OR adolescen*):ti) NOT ('practice guideline'/de OR (guideline*):ti)
