# Supplementary material for: Acute care pathway assessed through performance indicators during the COVID-19 pandemic in OECD countries (2020–2021): a scoping review
Source: BMC Emerg Med. 2024 Jan 26;24:19. doi: 10.1186/s12873-024-00938-7 (PMC10811879; doi:10.1186/s12873-024-00938-7)
Supplement: Supplementary file 4 — Additional file 4. List and characteristics of the studies included, from which indicators were extracted and collated. [file 12873_2024_938_MOESM4_ESM.pdf]

Additional file 4 – Characteristics of the studies included, from which indicators were extracted and collated (.xlsx)

Reference	Clinical area	Countries	Study design	Data source (1)	Data source (2)	Time period studied (Start)	Time period studied (End)
(Westgard et al. 2020)	Acute care utilisation	US	Retrospective cohort	Administrative data		15/02/2020	10/04/2020
(Baert et al. 2020)	Acute care utilisation	France	Retrospective cohort	Administrative data		01/03/2020	31-04-2020
(Cole et al. 2021)	Acute care utilisation	Canada	Retrospective cohort	Administrative data		01/02/2020	30/04/2020
(Ball et al. 2020)	Acute care utilisation	Australia	Retrospective cohort	Registry data	Administrative data	16/03/2020	12/05/2020
(Miyagami et al. 2021)	Acute care utilisation	Japan	Retrospective cohort	Administrative data		01/03/2020	31/05/2020
(Jain et al. 2021)	Acute care utilisation	US	Retrospective cohort	Administrative data		01/01/2020	30/06/2020
(Houshyar et al. 2020)	Acute care utilisation	US	Retrospective cohort	Clinical data		19/03/2020	02/04/2020
(Poyser et al. 2021)	Acute care utilisation	United Kingdom	Retrospective cohort	Clinical data	Registry data	24/03/2020	23/04/2020
(Işık and Çevik 2021)	Acute care utilisation	Turkey	Retrospective cohort	Administrative data		01/02/2020	31/07/2020
(Sekine et al. 2020)	Acute care utilisation	Japan	Retrospective cohort	Administrative data		01/01/2020	25/05/2020
(Gibson et al. 2020)	Acute care utilisation	US	Retrospective cohort	Administrative data		05/01/2020	30/05/2020
(Novara et al. 2020)	Acute care utilisation	Italy	Retrospective cohort	Administrative data		16/03/2020	22/03/2020
(Lim et al. 2020)	Acute care utilisation		Systematic review	Literature review			
(Borgmann et al. 2021)	Acute care utilisation	Germany	Retrospective cohort	Administrative data		12/03/2020	12/04/2020
(Lange et al. 2020)	Acute care utilisation	US	Retrospective cohort	Administrative data		05/01/2020	23/05/2020
(Franchini et al. 2021)	Acute care utilisation	Italy	Retrospective cohort	Administrative data		01/02/2020	31/03/2020
(Sharperson et al. 2021)	Acute care utilisation	US	Retrospective cohort	Clinical data		23/03/2020	27/04/2020

(Kuitunen et al. 2020)	Acute care utilisation	Finland	Retrospective cohort	Administrative data	01/02/2020	30/04/2020
(Goldberg et al. 2021)	Acute care utilisation	US	Retrospective cohort	Administrative data	15/02/2020	15/05/2020
(Madanelo et al. 2020)	Acute care utilisation	Portugal	Retrospective cohort	Administrative data	11/03/2020	01/04/2020
(Moon et al. 2020)	Acute care utilisation	US	Retrospective cohort	Administrative data	01/03/2020	30/04/2020
(Garrafa et al. 2020)	Acute care utilisation	Italy	Retrospective cohort	Registry data	01/02/2020	30/06/2020
(Mitchell et al. 2020)	Acute care utilisation	Australia	Retrospective cohort	Administrative data	26/03/2020	25/04/2020
(Griffith et al. 2021)	Acute care utilisation	US	Retrospective cohort	Administrative data	01/04/2020	30/04/2020
(Grasso, Massa, and Castelnuovo 2021)	Acute care utilisation	Italy	Retrospective cohort	Administrative data	15/02/2020	15/04/2020
(Fahrner, Bähler, and Lindner 2021)	Acute care utilisation	Switzerland	Retrospective cohort	Administrative data	14/03/2020	26/04/2020
(Isoletta et al. 2020)	Acute care utilisation	Italy	Retrospective cohort	Clinical data	22/02/2020	03/05/2020
(Deák, Fusz, and Kanizsai 2020)	Acute care utilisation	Hungary	Retrospective cohort	Survey data	01/04/2019	31/05/2020
(Kastritis et al. 2020)	Acute care utilisation	Greece	Retrospective cohort	Administrative data	01/03/2020	31/03/2020
(Şan et al. 2021)	Acute care utilisation	Turkey	Retrospective cohort	Administrative data	11/03/2020	24/04/2020
(Giannouchos et al. 2021)	Acute care utilisation	US	Retrospective cohort	Administrative data	01/01/2020	31/08/2020
(Walker et al. 2020)	Acute care utilisation	US	Retrospective cohort	Administrative data	09/02/2020	21/04/2020
(Rosell Ortiz et al. 2020)	Acute care utilisation	Spain	Retrospective cohort	Administrative data	01/02/2020	30/04/2020
(Agarwal et al. 2020)	Acute care utilisation	Canada	Retrospective cohort	Administrative data	12/03/2020	08/04/2020
(Porreca et al. 2020)	Acute care utilisation	Italy	Retrospective cohort	Administrative data	24/02/2020	15/03/2020
(Barten, Latten, and van Osch 2022)	Acute care utilisation	Netherlands	Retrospective cohort	Administrative data	15/02/2020	16/04/2020
(Boserup, McKenney, and Elkbuli 2020)	Acute care utilisation	US	Retrospective cohort	Administrative data	29/09/2019	05/04/2020

(Comelli, Scioscioli, and Cervellin 2020)	Acute care utilisation	Italy	Retrospective cohort	Administrative data	17/02/2020	12/04/2020
(Nopp et al. 2020)	Acute care utilisation	Austria	Retrospective cohort	Administrative data	01/01/2020	26/04/2020
(Hartnett et al. 2020)	Acute care utilisation	US	Retrospective cohort	Administrative data	29/03/2020	27/04/2020
(Franzolin et al. 2022)	Acute care utilisation	Italy	Retrospective cohort	Administrative data	10/02/2020	10/05/2020
(Anderson et al. 2022)	Acute care utilisation	Ireland	Retrospective cohort	Administrative data	16/03/2020	24/06/2020
(Kociejowski et al. 2021)	Acute care utilisation	United Kingdom	Retrospective cohort	Administrative data	23/03/2020	15/05/2020
(Görmeli Kurt and Çamcı 2021)	Acute care utilisation	Turkey	Retrospective cohort	Administrative data	01/03/2020	01/06/2020
(Motterle et al. 2021)	Acute care utilisation	Italy	Retrospective cohort	Administrative data	22/02/2020	30/03/2020
(Jeffery et al. 2020)	Acute care utilisation	US	Retrospective cohort	Administrative data	01/01/2020	30/04/2020
(Scquizzato et al. 2020)	Pre-hospital services		Systematic review	Literature review		
(Stöwhas and Lippert 2021)	Acute care utilisation	Germany	Retrospective cohort	Administrative data	07/03/2019	15/03/2020
(Burgard et al. 2021)	Acute care utilisation	Switzerland	Retrospective cohort	Administrative data	12/03/2019	06/06/2020
(Shreffler et al. 2021)	Acute care utilisation	US	Retrospective cohort	Clinical data	06/03/2020	25/06/2020
(Honeyford et al. 2021)	Acute care utilisation	United Kingdom	Retrospective cohort	Administrative data	06/01/2020	02/02/2020
(Long et al. 2022)	Acute care utilisation	US	Retrospective cohort	Administrative data	01/01/2020	30/11/2020
(Handberry et al. 2021)	Pre-hospital services	US	Retrospective cohort	Population-level data	01/01/2018	31/12/2020
(Lane et al. 2021)	Acute care utilisation	Canada	Retrospective cohort	Administrative data	01/12/2019	30/06/2020
(Inokuchi et al. 2021)	Pre-hospital services	Japan	Retrospective cohort	Administrative data	01/12/2019	30/04/2020
(Flamm, Lee, and Mencl 2022)	Acute care utilisation	US	Retrospective cohort	Administrative data	01/01/2020	31/07/2020
(Grunau et al. 2021)	Pre-hospital services	Canada	Retrospective cohort	Administrative data	15/03/2020	15/05/2020

(Burns et al. 2022)	Pre-hospital services	US	Retrospective cohort	Administrative data	Registry data	01/07/2020	28/02/2021
(Laukkanen et al. 2021)	Pre-hospital services	Finland	Retrospective cohort	Administrative data		01/03/2020	03/06/2020
(Chung et al. 2022)	Pre-hospital services	Korea	Retrospective cohort	Clinical data		01-01-2019	28-02-2021
(Wartelle et al. 2021)	Acute care utilisation	France	Retrospective cohort	Administrative data		16/02/2020	31/05/2020
(Mathew et al. 2021)	Pre-hospital services	US	Retrospective cohort	Registry data		03/10/2020	30/03/2020
(Lowe et al. 2021)	Acute care utilisation	US	Retrospective cohort	Administrative data		01/03/2020	30/06/2020
(Sless et al. 2021)	Acute care utilisation	Ireland	Retrospective cohort	Administrative data		15/02/2020	11/04/2020
(Venkatesh et al. 2021)	Acute care utilisation	US	Retrospective cohort	Registry data		01/01/2020	15/11/2020
(Rollman et al. 2021)	Pre-hospital services	US	Retrospective cohort	Registry data		01/02/2020	29/05/2020
(İlhan, Bozdereli Berikol, and Dogan 2021)	Acute care utilisation	Turkey	Retrospective cohort	Administrative data		01/04/2020	31/05/2020
(Müller et al. 2022)	Pre-hospital services	Germany	Retrospective cohort	Administrative data		22/03/2020	20/06/2020
(Reschen et al. 2021)	Acute care utilisation	United Kingdom	Retrospective cohort	Clinical data		17/03/2021	18/07/2021
(S. Lee et al. 2021)	Acute care utilisation	US	Retrospective cohort	Clinical data		01/03/2020	30/06/2020
(Bergrath et al. 2022)	Acute care utilisation	Germany	Retrospective cohort	Clinical data		20/01/2020	14/06/2020
(Garlisi et al. 2021)	Acute care utilisation	Italy	Retrospective cohort	Administrative data		12/01/2020	30/06/2020
(Montero-Pérez and Jiménez Murillo 2021)	Acute care utilisation	Spain	Retrospective cohort	Administrative data		14/03/2020	21/06/2020
(Wyatt et al. 2021)	Acute care utilisation	United Kingdom	Retrospective cohort	Administrative data		11/03/2020	30/06/2020
(Kiliç and Şendur 2021)	Acute care utilisation	Turkey	Retrospective cohort	Administrative data		01/04/2020	01/07/2020
(Grübl et al. 2021)	Pre-hospital services	Germany	Retrospective cohort	Administrative data		01/01/2020	31/05/2020
(Glober et al. 2021)	Pre-hospital services	US	Retrospective cohort	Clinical data		01/01/2020	30/06/2020

(Parikh et al. 2020)	Acute care utilisation	US	Retrospective cohort	Administrative data		11/04/2020	19/04/2020
(D'Ascenzi et al. 2021)	Pre-hospital services	Italy	Retrospective cohort	Administrative data		01/01/2020	31/03/2020
(Ferron et al. 2021)	Pre-hospital services	Canada	Retrospective cohort	Administrative data		02/01/2020	26/05/2020
(D. D. Lee et al. 2021)	Acute care utilisation	Canada	Retrospective cohort	Administrative data		17/03/2020	30/06/2020
(Rennert-May et al. 2021)	Acute care utilisation	Canada	Retrospective cohort	Administrative data		16/03/2020	23/09/2020
(Kalanj et al. 2021)	Acute care utilisation	Croatia	Retrospective cohort	Administrative data		01/01/2022	31/12/2020
(Russo et al. 2021)	Acute care utilisation	Italy	Retrospective cohort	Administrative data		10/03/2020	31/12/2020
(Kwok, Clapham, and Calder-Sprackman 2021)	Acute care utilisation	Canada	Retrospective cohort	Clinical data		11/03/2020	22/04/2020
(Muselli, Cofini, and Mammarella 2021)	Acute care utilisation	Italy	Retrospective cohort	Administrative data		09/03/2020	03/05/2020
(Alwood et al. 2021)	Acute care utilisation	US	Retrospective cohort	Clinical data		22/03/2020	31/12/2020
(Andrew et al. 2021)	Pre-hospital services	Australia	Retrospective cohort	Administrative data		16/03/2020	28/02/2021
(Vollmer et al. 2021)	Acute care utilisation	United Kingdom	Retrospective cohort	Administrative data		12/03/2020	31/05/2020
(Lucero et al. 2020)	Acute care utilisation	US	Retrospective cohort	Claims/billing data		16/03/2020	20/04/2020
(Bielski et al. 2021)	Acute care utilisation		Systematic review	Literature review			
(Nok et al. 2021)	Acute care utilisation	US	Retrospective cohort	Clinical data		01/01/2020	09/05/2020
(Daniels et al. 2021)	Acute care utilisation	United Kingdom	Retrospective cohort	Administrative data		01/03/2020	30/06/2020
(Nourian et al. 2022)	Acute care utilisation	US	Retrospective cohort	Clinical data		01/03/2020	30/06/2020
(Bardin et al. 2021)	Acute care utilisation	Italy	Retrospective cohort	Administrative data	Clinical data	01/01/2020	31/12/2020
(Soares et al. 2022)	Acute care utilisation	US	Retrospective cohort	Clinical data		01/01/2020	31/12/2020
(Jodal et al. 2022)	Acute care utilisation	Norway	Retrospective cohort	Clinical data		06/01/2020	31/05/2020

(Eppenberger, Golla, and Schmid 2022)	Acute care utilisation	Switzerland	Retrospective cohort	Clinical data		01/01/2020	31/12/2020
(DelPozo-Banos et al. 2022)	Acute care utilisation	United Kingdom	Retrospective cohort	Population-level data		02/01/2020	14/03/2021
(Antonazzo et al. 2021)	Acute care utilisation	Italy	Retrospective cohort	Administrative data		01/01/2020	27/12/2020
(Jessup et al. 2021)	Acute care utilisation	Australia	Retrospective cohort	Administrative data		16/03/2020	30/09/2020
(Lawless, Burgess, and Bourke 2022)	Acute care utilisation	United Kingdom	Retrospective cohort	Administrative data	Clinical data	26/03/2020	31/12/2020
(Ghaderi et al. 2021)	Acute care utilisation	US	Retrospective cohort	Clinical data	Administrative data	01/01/2020	31/08/2020
(Barış et al. 2022)	Acute care utilisation	Turkey	Retrospective cohort	Clinical data		11/03/2020	31/05/2020
(Domont, P., Debaize, S., and Curac, S. 2022)	Pre-hospital services	Belgium	Retrospective cohort	Administrative data		01/02/2020	31/12/2020
(Loftus et al. 2022)	Acute care utilisation	US	Retrospective cohort	Administrative data		08/03/2020	27/02/2021
(Lopez-Villegas et al. 2022)	Acute care utilisation	Spain	Retrospective cohort	Administrative data		01/01/2020	31/12/2020
(Meurice et al. 2021)	Acute care utilisation	France	Retrospective cohort	Administrative data		09/03/2020	20/12/2020
(Vest et al. 2022)	Acute care utilisation	France	Retrospective cohort	Administrative data	Clinical data	17/02/2020	27/04/2020
(Sarc et al. 2022)	Acute care utilisation	Slovenia	Retrospective cohort	Population-level data	Claims/billing data	01/01/2020	28/02/2021
(Damjanovic et al. 2022)	Pre-hospital services	Germany	Retrospective cohort	Administrative data		27/02/2020	30/04/2020
(Giusti et al. 2022)	Acute care utilisation	Italy	Retrospective cohort	Administrative data		01/02/2020	31/10/2020
(Konson et al. 2022)	Acute care utilisation	Israel	Retrospective cohort	Clinical data		01/01/2020	31/12/2020
(Inglin et al. 2022)	Acute care utilisation	Finland	Retrospective cohort	Clinical data		16/03/2020	31/12/2020
(Baugh et al. 2021)	Acute care utilisation	US	Retrospective cohort	Clinical data		01/03/2020	30/04/2020
(Haklai et al. 2022)	Acute care utilisation	Israel	Retrospective cohort	Clinical data		01/01/2020	31/12/2020
(Chavez et al. 2022)	Pre-hospital services	US	Retrospective cohort	Registry data		11/03/2020	31/12/2020

(Sagy et al. 2022)	Acute care utilisation	Israel	Retrospective cohort	Administrative data		01/03/2020	30/04/2021
(Başer and Başer 2021)	Acute care utilisation	Turkey	Retrospective cohort	Administrative data		01/04/2020	31-11-2020
(Musajee et al. 2022)	Acute care utilisation	United Kingdom	Prospective cohort	Clinical data		15/03/2020	30/05/2020
(Lastrucci et al. 2022)	Acute care utilisation	Italy	Retrospective cohort	Administrative data		01/01/2020	28/06/2020
(Zeppieri, Maria Letizia Salvetat, and Salati 2021)	Acute care utilisation	Italy	Retrospective cohort	Clinical data		01/01/2020	30/04/2020
(Masuda et al. 2022)	Pre-hospital services		Systematic review	Literature review			
(Maclagan et al. 2022)	Acute care utilisation	Canada	Retrospective cohort	Administrative data		01/03/2020	28/02/2021
(O'Brien et al. 2020)	Acute general surgery	Canada	Retrospective cohort	Clinical data		15/03/2020	15/04/2020
(Nishida, Otagiri, and Tauchi 2021)	Acute general surgery	Japan	Retrospective cohort	Clinical data		01/03/2020	30/06/2020
(Bajomo et al. 2021)	Acute general surgery	UK	Prospective cohort	Clinical data		14/03/2020	14/05/2020
(Aviran et al. 2020)	Acute general surgery	Israel	Prospective cohort	Administrative data		15/03/2020	14/04/2020
(Köhler et al. 2021)	Acute general surgery	Germany	Retrospective cohort	Claims data		01/03/2020	30/06/2020
(O'Connell et al. 2021)	Acute general surgery	Ireland	Retrospective cohort	Clinical data		01/03/2020	30/04/2020
(McGuinness and Hsee 2020)	Acute general surgery	New Zealand	Retrospective cohort	Clinical data		26/03/2020	27/04/2020
(Dick et al. 2020)	Acute general surgery	Scotland	Prospective cohort	Clinical data		23/03/2020	07/05/2020
(Gomez et al. 2021)	Acute general surgery	Canada	Retrospective cohort	Administrative data	Registry data	11/03/2020	30/06/2020
(Antakia et al. 2021)	Acute general surgery	UK	Prospective cohort	Clinical data		10/03/2020	05/07/2020
(Aboul-Enein et al. 2021)	Acute general surgery	UK	Retrospective cohort	Clinical data		18/05/2020	31/05/2020
(Vallès et al. 2021)	Acute general surgery	US	Retrospective cohort	Administrative data		01/01/2020	31/05/2020

(Karlafti et al. 2021)	Acute general surgery	Greece	Retrospective cohort	Clinical data		01/03/2020	28/02/2021
(Lund et al. 2021)	Acute general surgery	US	Retrospective cohort	Clinical data		01/03/2020	31/05/2020
(Farber et al. 2021)	Acute general surgery	US	Retrospective cohort	Clinical data		01/03/2020	30/06/2020
(Kurihara et al. 2021)	Acute general surgery	Italy	Survey	Survey data		21/02/2020	03/04/2020
(Amendola et al. 2021)	Acute general surgery	Italy	Retrospective cohort	Clinical data		09/03/2020	27/04/2020
(Callan, Assaf, and Bevan 2020)	Acute general surgery	UK	Retrospective cohort	Clinical data		23/03/2020	05/04/2020
(Rajesh et al. 2022)	Acute general surgery	Ireland	Retrospective cohort	Administrative data		29/02/2020	31-11-2020
(Vissio et al. 2021)	Acute general surgery	Italy	Retrospective cohort	Clinical data		01/03/2020	31/12/2020
(Ma et al. 2022)	Acute general surgery	Australia	Retrospective cohort	Clinical data		01/03/2020	28/02/2021
(Rudnicki et al. 2022)	Acute general surgery	Israel	Retrospective cohort	Clinical data		01/03/2020	30/04/2020
(Siegel et al. 2022)	Acute general surgery	Germany	Retrospective cohort	Claims data		02/03/2020	20/06/2021
(Lapsekili, Buldanlı, and Peker 2021)	Acute general surgery	Turkey	Retrospective cohort	Clinical data		11/03/2020	31/12/2020
(Derebey et al. 2022)	Acute general surgery	Turkey	Retrospective cohort	Clinical data		11/03/2020	10/03/2021
(Hossain et al. 2022)	Acute general surgery	UK	Retrospective cohort	Clinical data		01/03/2020	30/06/2020
(Reinke et al. 2023)	Acute general surgery	US	Retrospective cohort	Administrative data	Clinical data	17/03/2020	17/02/2021
(Balvardi et al. 2022)	Acute general surgery	Canada	Retrospective cohort	Administrative data	Clinical data	13/03/2020	13/05/2020

Aboul-Enein, Mohamed Saad, Peter Ishak, Maninder Bhambra, Pol Ricart, Lisa Al-makdase, Moustafa Mourad, Martin Wadley, and Anthony Perry. 2021. 'At a Glance: The Impact of COVID-19 on Emergency General Surgery'. *Surgical Practice* 25 (1): 16–24. https://doi.org/10.1111/1744-1633.12475.

Agarwal, Minu, Amar Udare, Abdullah Alabousi, Christian B. van der Pol, Lucas Ramonas, Ken Mascola, Britney Edmonds, and Milita Ramonas. 2020. 'Impact of the COVID-19 Pandemic on Emergency CT Head Utilization in Ontario—an Observational Study of Tertiary Academic Hospitals'. *Emergency Radiology* 27 (6): 791–97. https://doi.org/10.1007/s10140-020-01857-3.

Alwood, Shannon, Mandi W. Musso, Glenn N. Jones, Joel Mosley, Brittany Wippel, Lauren Theriot, and Diana Hamer. 2021. 'The Impact of the COVID-19 Pandemic and Governor Mandated Stay at Home Order on Emergency Department Super Utilizers'. *The American Journal of Emergency Medicine* 48 (October): 114–19. https://doi.org/10.1016/j.ajem.2021.04.022.

Amendola, Alfonso, Giuseppe Palomba, Maria Gaudiello, Vincenza Paola Dinuzzi, Ester Marra, Ferdinando Fusco, Michele Lanza, et al. 2021. 'Impact of SARS-Cov-2 Pandemic on Emergency General Surgery. A Single-Center Observational Study'. *Annali Italiani Di Chirurgia* 92: 317–22.

Anderson, Steven, Daniel McNicholas, Claudine Murphy, Ijaz Cheema, Liza McLornan, Niall Davis, and Mark Quinlan. 2022. 'The Impact of COVID-19 on Acute Urinary Stone Presentations: A Single-Centre Experience'. *Irish Journal of Medical Science (1971 -)* 191 (1): 45–49. https://doi.org/10.1007/s11845-021-02562-x.

Andrew, Emily, Ziad Nehme, Michael Stephenson, Tony Walker, and Karen Smith. 2021. 'The Impact of the COVID-19 Pandemic on Demand for Emergency Ambulances in Victoria, Australia'. *Prehospital Emergency Care*, July, 1–7. https://doi.org/10.1080/10903127.2021.1944409.

Antakia, Ramez, Athanasios Xanthis, Fanourios Georgiades, Victoria Hudson, James Ashcroft, Siobhan Rooney, Aminder A. Singh, et al. 2021. 'Acute Appendicitis Management during the COVID-19 Pandemic: A Prospective Cohort Study from a Large UK Centre'. *International Journal of Surgery* 86 (February): 32–37. https://doi.org/10.1016/j.ijsu.2020.12.009.

Antonazzo, Ippazio Cosimo, Carla Fornari, Sandy Maumus-Robert, Eleonora Cei, Olga Paoletti, Sara Conti, Paolo Angelo Cortesi, Lorenzo Giovanni Mantovani, Rosa Gini, and Giampiero Mazzaglia. 2021. 'Impact of COVID-19 Lockdown, during the Two Waves, on Drug Use and Emergency Department Access in People with Epilepsy: An Interrupted Time-Series Analysis'. *International Journal of Environmental Research and Public Health* 18 (24): 13253. https://doi.org/10.3390/ijerph182413253.

Aviran, Eyal, Shachar Laks, Haggai Benvenisti, Saed Khalilieh, Dan Assaf, Nimrod Aviran, David Hazzan, et al. 2020. 'The Impact of the COVID-19 Pandemic on General Surgery Acute Admissions and Urgent Operations: A Comparative Prospective Study'. *The Israel Medical Association Journal: IMAJ* 11 (22): 673–79.

Baert, Valentine, Deborah Jaeger, Hervé Hubert, Jean-Baptiste Lascarrou, Guillaume Debaty, Tahar Chouihed, and François Javaudin. 2020. 'Assessment of Changes in Cardiopulmonary Resuscitation Practices and Outcomes on 1005 Victims of Out-of-Hospital Cardiac Arrest during the COVID-19 Outbreak: Registry-Based Study'. *Scandinavian Journal of Trauma, Resuscitation and Emergency Medicine* 28 (1): 119. https://doi.org/10.1186/s13049-020-00813-x.

Bajomo, Oreoluwa, Rumneek Hampal, Paul Sykes, and Anur Miah. 2021. 'Managing Appendicitis during the COVID-19 Era: A Single Centre Experience & Implications for Future Practice'. *Annals of Medicine and Surgery* 63 (March): 102168. https://doi.org/10.1016/j.amsu.2021.02.014.

Ball, J., Z. Nehme, S. Bernard, D. Stub, M. Stephenson, and K. Smith. 2020. 'Collateral Damage: Hidden Impact of the COVID-19 Pandemic on the out-of-Hospital Cardiac Arrest System-of-Care'. *Resuscitation* 156: 157–63.

Balvardi, Saba, Josie Cipolla, Nawar Touma, Tharaniya Vallipuram, Natasha Barone, Reginold Sivarajan, Pepa Kaneva, et al. 2022. 'Impact of the Covid-19 Pandemic on Rates of Emergency Department Utilization and Hospital Admission Due to General Surgery Conditions'. *Surgical Endoscopy* 36 (9): 6751–59. https://doi.org/10.1007/s00464-021-08956-3.

Bardin, Andrea, Alessandra Buja, Claudio Barbiellini Amidei, Matteo Paganini, Andrea Favaro, Mario Saia, and Vincenzo Baldo. 2021. 'Elderly People's Access to Emergency Departments during the COVID-19 Pandemic: Results from a Large Population-Based Study in Italy'. *Journal of Clinical Medicine* 10 (23): 5563. https://doi.org/10.3390/jcm10235563.

Barış, Mine Esen, Mukaddes Damla Çiftçi, Suzan Güven Yılmaz, and Halil Ateş. 2022. 'Impact of COVID-19-Related Lockdown on Glaucoma Patients'. *Turkish Journal of Ophthalmology* 52 (2): 91–95. https://doi.org/10.4274/tjo.galenos.2021.83765.

Barten, Dennis G., Gideon H.P. Latten, and Frits H.M. van Osch. 2022. 'Reduced Emergency Department Utilization During the Early Phase of the COVID-19 Pandemic: Viral Fear or Lockdown Effect?' *Disaster Medicine and Public Health Preparedness* 16 (1): 36–39. https://doi.org/10.1017/dmp.2020.303.

Başer, Hülya Yılmaz, and Aykut Başer. 2021. 'The Impact of the Covid-19 Pandemic on the Short and Mid-Term Urological Emergencies and the Emergency Department'. *Journal of Experimental and Clinical Medicine* 38 (4): 466–70. https://doi.org/10.52142/omujecm.38.4.12.

Baugh, Joshua J., Benjamin A. White, Dustin McEvoy, Brian J. Yun, David F.M. Brown, Ali S. Raja, and Sayon Dutta. 2021. 'The Cases Not Seen: Patterns of Emergency Department Visits and Procedures in the Era of COVID-19'. *The American Journal of Emergency Medicine* 46 (August): 476–81. https://doi.org/10.1016/j.ajem.2020.10.081.

Bergrath, Sebastian, Tobias Strapatsas, Michael Tuemen, Thorsten Reith, Marc Deussen, Olaf Aretz, Andreas Hohn, and Andreas Lahm. 2022. 'Impact of the COVID-19 Pandemic on Emergency Medical Resources: An Observational Multicenter Study Including All Hospitals in a Major Urban Center of the Rhein-Ruhr Metropolitan Region'. *Die Anaesthesiologie* 71 (S2): 171–79. https://doi.org/10.1007/s00101-021-01005-7.

Bielski, Karol, Agnieszka Szarpak, Miłosz Jaroslaw Jaguszewski, Tomasz Kopiec, Jacek Smereka, Aleksandra Gasecka, Przemysław Wolak, et al. 2021. 'The Influence of COVID-19 on Out-Hospital Cardiac Arrest Survival Outcomes: An Updated Systematic Review and Meta-Analysis'. *Journal of Clinical Medicine* 10 (23): 5573. https://doi.org/10.3390/jcm10235573.

Borgmann, Hendrik, Julian P. Struck, Angelika Mattigk, Mike Wenzel, Adrian Pilatz, Jennifer Kranz, Richard Weiten, Nicolas Von Landenberg, Philipp Julian Spachmann, and Cem Aksoy. 2021. 'Increased Severe Adverse Outcomes and Decreased Emergency Room Visits for Pyelonephritis: First Report of Collateral Damage during COVID-19 Pandemic in Urology'. *Urologia Internationalis* 105 (3–4): 199–205.

Boserup, Brad, Mark McKenney, and Adel Elkbuli. 2020. 'The Impact of the COVID-19 Pandemic on Emergency Department Visits and Patient Safety in the United States'. *The American Journal of Emergency Medicine* 38 (9): 1732–36. https://doi.org/10.1016/j.ajem.2020.06.007.

Burgard, Marie, Floryn Cherbanyk, Konstantinos Nassiopoulos, Sonaz Malekzadeh, François Pugin, and Bernhard Egger. 2021. 'An Effect of the COVID-19 Pandemic: Significantly More Complicated Appendicitis Due to Delayed Presentation of Patients!' Edited by Robert Jeenchen Chen. *PLOS ONE* 16 (5): e0249171. https://doi.org/10.1371/journal.pone.0249171.

Burns, Timothy A., Christopher Touzeau, Benjamin T. Kaufman, Alan L. Butsch, Roumen Vesselinov, and Roger M. Stone. 2022. 'Decreases in out of Hospital Cardiac Arrest (OHCA) Outcome Metrics Persist When Known COVID Patients Are Excluded from Analysis'. *The American Journal of Emergency Medicine* 51 (January): 64–68. https://doi.org/10.1016/j.ajem.2021.09.083.

Callan, Rory, Nazrin Assaf, and Katharine Bevan. 2020. 'Impact of the COVID-19 Pandemic on Acute General Surgical Admissions in a District General Hospital in the United Kingdom: A Retrospective Cohort Study'. *Surgery Research and Practice* 2020 (August): 1–7. https://doi.org/10.1155/2020/2975089.

Chavez, Summer, Ryan Huebinger, Hei Kit Chan, Joseph Gill, Lynn White, Donna Mendez, Jeffrey L. Jarvis, et al. 2022. 'The Impact of COVID-19 on Incidence and Outcomes from out-of-Hospital Cardiac Arrest (OHCA) in Texas'. *The American Journal of Emergency Medicine* 57 (July): 1–5. https://doi.org/10.1016/j.ajem.2022.04.006.

Chung, Hosub, Myeong Namgung, Dong Hoon Lee, Yoon Hee Choi, and Sung Jin Bae. 2022. 'Effect of Delayed Transport on Clinical Outcomes among Patients with Cardiac Arrest during the Coronavirus Disease 2019 Pandemic'. *Australasian Emergency Care* 25 (3): 241–46. https://doi.org/10.1016/j.auec.2021.11.006.

Cole, Valancy, Paul Atkinson, Robert Hanlon, Daniel J. Dutton, Tong Liu, Jacqueline Fraser, David Lewis, Keith R. Brunt, Hana Wiemer, and Tara Dahn. 2021. 'CO-AVoID: Coronavirus Outbreak Affecting Variability of Presentations to a Local Emergency Department'. *Canadian Journal of Emergency Medicine* 23: 232–36.

Comelli, Ivan, Francesco Scioscioli, and Gianfranco Cervellin. 2020. 'Impact of the COVID-19 Epidemic on Census, Organization and Activity of a Large Urban Emergency Department.: COVID-19 Epidemic in a Large Emergency Department'. *Acta Bio Medica Atenei Parmensis* 91 (2): 45–49. https://doi.org/10.23750/abm.v91i2.9565.

Damjanovic, Domagoj, Jan-Steffen Pooth, Rebecca Steger, Martin Boeker, Michael Steger, Julian Ganter, Tobias Hack, et al. 2022. 'Observational Study on Implications of the COVID-19-Pandemic for Cardiopulmonary Resuscitation in out-of-Hospital Cardiac Arrest: Qualitative and Quantitative Insights from a Model Region in Germany'. *BMC Emergency Medicine* 22 (1): 85. https://doi.org/10.1186/s12873-022-00628-2.

Daniels, Natasha F, Raiiq Ridwan, Ed BG Barnard, Talha M Amanullah, and Catherine Hayhurst. 2021. 'A Comparison of Emergency Department Presentations for Medically Unexplained Symptoms in Frequent Attenders during COVID-19'. *Clinical Medicine* 21 (4): e399–402. https://doi.org/10.7861/clinmed.2020-1093.

D'Ascenzi, Flavio, Matteo Cameli, Silvia Forni, Fabrizio Gemmi, Claudia Szasz, Valeria Di Fabrizio, Maria Teresa Mechi, Matteo Nocci, Sergio Mondillo, and Serafina Valente. 2021. 'Reduction of Emergency Calls and Hospitalizations for Cardiac Causes: Effects of Covid-19 Pandemic and Lockdown in Tuscany Region'. *Frontiers in Cardiovascular Medicine* 8 (March): 625569. https://doi.org/10.3389/fcvm.2021.625569.

Deák, András, Katalin Fusz, and Péter Kanizsai. 2020. 'Patient Satisfaction during the COVID-19 Pandemic in an Emergency Department'. *Orvosi Hetilap* 161 (43): 1819–23. https://doi.org/10.1556/650.2020.32037.

DelPozo-Banos, M., S. C. Lee, Y. Friedmann, A. Akbari, F. Torabi, K. Lloyd, R. A. Lyons, and A. John. 2022. 'Healthcare Contacts with Self-Harm during COVID-19: An e-Cohort Whole-Population-Based Study Using Individual-Level Linked Routine Electronic Health Records in Wales, UK, 2016—March 2021'. Edited by Michelle Torok. *PLOS ONE* 17 (4): e0266967. https://doi.org/10.1371/journal.pone.0266967.

Derebey, Murat, Mahmut Arif Yüksek, Ufuk Karabacak, Vahit Mutlu, Salih Raşit MiZan, İsmail Alper Tarim, Aysu Başak Özbalci, et al. 2022. 'Effects of COVID-19 Pandemic on Management of Acute Cholecystitis: A Single Tertiary Center's Experience'. *Journal of Experimental and Clinical Medicine* 39 (1): 164–68. https://doi.org/10.52142/omujecm.39.1.33.

Dick, Lachlan, James Green, Jasmine Brown, Ewan Kennedy, Richard Cassidy, Salasiah Othman, and Martin Berlansky. 2020. 'Changes in Emergency General Surgery During Covid-19 in Scotland: A Prospective Cohort Study'. *World Journal of Surgery* 44 (11): 3590–94. https://doi.org/10.1007/s00268-020-05760-3.

Domont, P., Debaize, S., and Curac, S. 2022. 'Impact Du SARS-CoV-2 Sur Les Arrêts Cardiaques Extrahospitaliers.' *Revue Medicale de Bruxelles* 43: 110–16. https://doi.org/10.30637/2022.21-021.

Eppenberger, Leila Sara, Kathrin Golla, and Martin K. Schmid. 2022. 'Emergency Vitrectomies for Retinal Detachment Before and During the Coronavirus Pandemic – A Retrospective Single Centre Analysis'. *Klinische Monatsblätter für Augenheilkunde* 239 (04): 476–83. https://doi.org/10.1055/a-1808-6258.

Fahrner, René, Stefan Bähler, and Gregor Lindner. 2021. 'COVID-19 Lock-down Significantly Reduced Number of Surgical Presentations in an Emergency Department'. *Wiener Klinische Wochenschrift* 133 (7–8): 399–402. https://doi.org/10.1007/s00508-021-01810-5.

Farber, Orly Nadell, Giselle I Gomez, Ashley L Titan, Andrea T Fisher, Christopher J Puntasecca, Veronica Toro Arana, Arielle Kempinsky, et al. 2021. 'Impact of COVID-19 on Presentation, Management, and Outcomes of Acute Care Surgery for Gallbladder Disease and Acute Appendicitis'. *World Journal of Gastrointestinal Surgery* 13 (8): 859–70. https://doi.org/10.4240/wjgs.v13.i8.859.

Ferron, Richard, Gina Agarwal, Rhiannon Cooper, and Douglas Munkley. 2021. 'The Effect of COVID-19 on Emergency Medical Service Call Volumes and Patient Acuity: A Cross-Sectional Study in Niagara, Ontario'. *BMC Emergency Medicine* 21 (1): 39. https://doi.org/10.1186/s12873-021-00431-5.

Flamm, Avram, Alexander Lee, and Francis Mencl. 2022. 'COVID-19: A Comprehensive Analysis of the Pandemic's Effect on an Emergency Department'. *Disaster Medicine and Public Health Preparedness* 16 (6): 2367–70. https://doi.org/10.1017/dmp.2021.182.

Franchini, Stefano, Marzia Spessot, Giovanni Landoni, Cecilia Piani, Chiara Cappelletti, Federica Mariani, Simona Mauri, Maria Vittoria Taglietti, Manuela Fortunato, and Federico Furlan. 2021. 'Stranger Months: How SARS-CoV-2, Fear of Contagion, and Lockdown Measures Impacted Attendance and Clinical Activity during February and March 2020 at an Urban Emergency Department in Milan'. *Disaster Medicine and Public Health Preparedness* 15 (5): e33–42.

Franzolin, Elia, Stefano Casati, Ottavia Albertini, Giulio Antonelli, Pierpaolo Marchetti, Antonio Bonora, and Giorgio Marchini. 2022. 'Impact of Covid-19 Pandemic on Ophthalmic Emergency Department in an Italian Tertiary Eye Centre'. *European Journal of Ophthalmology* 32 (1): 680–87. https://doi.org/10.1177/1120672121998223.

Garlisi, C., D. Licandro, A. Siani, S. Rodolfi, S. Pansini, L. I. Garcia Navarro, A. Carriero, G. C. Avanzi, and L. M. Castello. 2021. 'Impact of the COVID-19 Pandemic on the Activity of the Radiological Emergency Department: The Experience of the Maggiore Della Carità Hospital in Novara'. *Emergency Radiology* 28 (4): 705–11. https://doi.org/10.1007/s10140-021-01928-z.

Garrafa, Emirena, Rosella Levaggi, Raffaele Miniaci, and Ciro Paolillo. 2020. ‘When Fear Backfires: Emergency Department Accesses during the Covid-19 Pandemic’. *Health Policy* 124 (12): 1333–39.

Ghaderi, Hamid, Jeffrey R. Stowell, Murtaza Akhter, Craig Norquist, Paul Pugsley, and Vignesh Subbian. 2021. ‘Impact of COVID-19 Pandemic on Emergency Department Visits: A Regional Case Study of Informatics Challenges and Opportunities’. *AMIA ... Annual Symposium Proceedings. AMIA Symposium* 2021: 496–505.

Giannouchos, Theodoros V., Joseph Biskupiak, Michael J. Moss, Diana Brixner, Elena Andreyeva, and Benjamin Ukert. 2021. ‘Trends in Outpatient Emergency Department Visits during the COVID-19 Pandemic at a Large, Urban, Academic Hospital System’. *The American Journal of Emergency Medicine* 40 (February): 20–26. https://doi.org/10.1016/j.ajem.2020.12.009.

Gibson, Averi L., Byron Y. Chen, Max P. Rosen, S. Nicolas Paez, and Hao S. Lo. 2020. ‘Impact of the COVID-19 Pandemic on Emergency Department CT for Suspected Diverticulitis’. *Emergency Radiology* 27: 773–80.

Giusti, Gian Domenico, Maria Rosaria Cozzolino, Alessio Gili, Andrea Ceccagnoli, Monia Ceccarelli, Paolo Groff, and Nicola Ramacciati. 2022. ‘Patients Who Leave the Emergency Department without Being Seen. Has COVID-19 Affected This Phenomenon?’ *Acta Biomedica Atenei Parmensis* 93 (S2): e2022188. https://doi.org/10.23750/abm.v93iS2.12392.

Glober, Nancy K., Michael Supples, Greg Faris, Thomas Arkins, Shawn Christopher, Tyler Fulks, David Rayburn, et al. 2021. ‘Out-of-Hospital Cardiac Arrest Volumes and Characteristics during the COVID-19 Pandemic’. *The American Journal of Emergency Medicine* 48 (October): 191–97. https://doi.org/10.1016/j.ajem.2021.04.072.

Goldberg, Scott A., Rebecca E. Cash, Gregory Peters, Scott G. Weiner, P. Gregg Greenough, and Raghu Seethala. 2021. ‘The Impact of COVID-19 on Statewide EMS Use for Cardiac Emergencies and Stroke in Massachusetts’. *Journal of the American College of Emergency Physicians Open* 2 (1): e12351.

Gomez, David, Andrea N. Simpson, Colin Sue-Chue-Lam, Charles de Mestral, Fahima Dossa, Jordan Nantais, Andrew S. Wilton, David Urbach, Peter C. Austin, and Nancy N. Baxter. 2021. ‘A Population-Based Analysis of the Impact of the COVID-19 Pandemic on Common Abdominal and Gynecological Emergency Department Visits’. *Canadian Medical Association Journal* 193 (21): E753–60. https://doi.org/10.1503/cmaj.202821.

Görmeli Kurt, Nazlı, and Melih Çamcı. 2021. ‘COVID-19: How Do Emergency Departments Fare after Normalisation Steps?’ *International Journal of Clinical Practice* 75 (2). https://doi.org/10.1111/ijcp.13912.

Grasso, Angelica A.C., Guido Massa, and Mauro Castelnuovo. 2021. ‘The Impact of COVID-19 Pandemic on Urological Emergencies: A Multicenter Experience on over 3,000 Patients’. *Urologia Internationalis* 105 (1–2): 17–20. https://doi.org/10.1159/000511757.

Griffith, April M., Patrick Ockerse, Akram Shaaban, and Christopher Kelly. 2021. ‘Effect of the COVID-19 Pandemic on CT Scans Ordered from the Emergency Department for Abdominal Complaints’. *Emergency Radiology* 28 (3): 485–95. https://doi.org/10.1007/s10140-021-01907-4.

Grübl, T., B. Plöger, M. C. Sassen, A. Jerrentrup, B. Schieffer, and S. Betz. 2021. ‘Prähospitaler Kreislaufstillstand im Lockdown: Auswirkungen der übergreifenden Infektionspräventionsmaßnahmen während der ersten SARS-CoV-2-Welle (Out-of-hospital cardiac arrest during lockdown)’. *Notfall & Rettungsmedizin*, 1–6. https://doi.org/10.1007/s10049-021-00932-7.

Grunau, Brian, Jennie Helmer, Sung Lee, Joe Acker, Jon Deakin, Richard Armour, John Tallon, Sandra Jenneson, Jim Christenson, and Frank X. Scheuermeyer. 2021. 'Decrease in Emergency Medical Services Utilization during Early Stages of the COVID-19 Pandemic in British Columbia'. *Canadian Journal of Emergency Medicine* 23 (2): 237–41. https://doi.org/10.1007/s43678-020-00062-y.

Haklai, Ziona, Yael Applbaum, Vicki Myers, Mor Saban, Ethel-Sherry Gordon, Osnat Luxenburg, and Rachel Wilf-Miron. 2022. 'The Effect of the COVID-19 Pandemic on Non-COVID Respiratory ED Visits in Israel'. *The American Journal of Emergency Medicine* 53 (March): 215–21. https://doi.org/10.1016/j.ajem.2022.01.005.

Handberry, Maya, Lara Bull-Otterson, Mengtao Dai, N Clay Mann, Eric Chaney, Jeff Ratto, Kalanthe Horiuchi, et al. 2021. 'Changes in Emergency Medical Services Before and During the COVID-19 Pandemic in the United States, January 2018–December 2020'. *Clinical Infectious Diseases* 73 (Supplement_1): S84–91. https://doi.org/10.1093/cid/ciab373.

Hartnett, Kathleen P., Aaron Kite-Powell, Jourdan DeVies, Michael A. Coletta, Tegan K. Boehmer, Jennifer Adjemian, Adi V. Gundlapalli, and National Syndromic Surveillance Program Community of Practice. 2020. 'Impact of the COVID-19 Pandemic on Emergency Department Visits — United States, January 1, 2019–May 30, 2020'. *MMWR. Morbidity and Mortality Weekly Report* 69 (23): 699–704. https://doi.org/10.15585/mmwr.mm6923e1.

Honeyford, Kate, Charles Coughlan, Ruud Nijman, Paul Expert, Gabriel Burcea, Ian Maconochie, Anne Kinderlerer, Graham Cooke, and Ceire Costelloe. 2021. 'Changes in Emergency Department Activity and the First COVID-19 Lockdown: A Cross-Sectional Study'. *Western Journal of Emergency Medicine* 22 (3). https://doi.org/10.5811/westjem.2021.2.49614.

Hossain, Naveed, Vishnu Naidu, Shady Hosny, Mohamed Khalifa, Pawan Mathur, and Maitham Al Whouhayb. 2022. 'Hospital Presentations of Acute Diverticulitis During COVID-19 Pandemic May Be More Likely to Require Surgery Due to Increased Severity: A Single-Centre Experience'. *The American Surgeon* 88 (1): 133–39. https://doi.org/10.1177/0003134820982560.

Houshyar, Roozbeh, Karen Tran-Harding, Justin Glavis-Bloom, Michael Nguyentat, John Mongan, Chantal Chahine, Thomas W. Loehfelm, Marc D. Kohli, Edward J. Zaragoza, and Paul M. Murphy. 2020. 'Effect of Shelter-in-Place on Emergency Department Radiology Volumes during the COVID-19 Pandemic'. *Emergency Radiology* 27: 781–84.

İlhan, Buğra, Göksu Bozdereli Berikol, and Halil Dogan. 2021. 'Impact of COVID-19 Outbreak on Emergency Visits and Emergency Consultations: A Cross-Sectional Study'. *Cureus* 13 (3): e14052. https://doi.org/10.7759/cureus.14052.

Inglin, Laura, Katja Wikström, Marja-Leena Lamidi, and Tiina Laatikainen. 2022. 'The Adverse Effect of the COVID-19 Pandemic on Health Service Usage among Patients with Type 2 Diabetes in North Karelia, Finland'. *BMC Health Services Research* 22 (1): 725. https://doi.org/10.1186/s12913-022-08105-z.

Inokuchi, Ryota, Kojiro Morita, Masao Iwagami, Taeko Watanabe, Masatoshi Ishikawa, and Nanako Tamiya. 2021. 'Changes in the Proportion and Severity of Patients with Fever or Common Cold Symptoms Utilizing an After-Hours House Call Medical Service during the COVID-19 Pandemic in Tokyo, Japan: A Retrospective Cohort Study'. *BMC Emergency Medicine* 21 (1): 64. https://doi.org/10.1186/s12873-021-00458-8.

Işık, Gülşah Çıkrıkçı, and Yunsur Çevik. 2021. 'Impact of COVID-19 Pandemic on Visits of an Urban Emergency Department'. *The American Journal of Emergency Medicine* 42: 78–82.

Isoletta, Eugenio, Camilla Vassallo, Valeria Brazzelli, Chiara Giorgini, Carlo Francesco Tomasini, Anna Sabena, Stefano Perlini, Annalisa De Silvestri, and Stefania Barruscotti. 2020. ‘Emergency Accesses in Dermatology Department during the Covid-19 Pandemic in a Referral Third Level Center in the North of Italy’. *Dermatologic Therapy* 33 (6). https://doi.org/10.1111/dth.14027.

Jain, Nikhil, Michael Berkenbush, David C. Feldman, Barnet Eskin, and John R. Allegra. 2021. ‘Effect of COVID19 on Prehospital Pronouncements and ED Visits for Stroke and Myocardial Infarction’. *The American Journal of Emergency Medicine* 43: 46–49.

Jeffery, Molly M., Gail D’Onofrio, Hyung Paek, Timothy F. Platts-Mills, William E. Soares, Jason A. Hoppe, Nicholas Genes, Bidisha Nath, and Edward R. Melnick. 2020. ‘Trends in Emergency Department Visits and Hospital Admissions in Health Care Systems in 5 States in the First Months of the COVID-19 Pandemic in the US’. *JAMA Internal Medicine* 180 (10): 1328. https://doi.org/10.1001/jamainternmed.2020.3288.

Jessup, Rebecca Leigh, C Bramston, A Beauchamp, A Gust, N Cvetanovska, Y Cao, C Haywood, et al. 2021. ‘Impact of COVID-19 on Emergency Department Attendance in an Australia Hospital: A Parallel Convergent Mixed Methods Study’. *BMJ Open* 11 (12): e049222. https://doi.org/10.1136/bmjopen-2021-049222.

Jodal, Henriette C., Frederik E. Juul, Ishita Barua, Michael Bretthauer, Mette Kalager, Magnus Løberg, and Louise Emilsson. 2022. ‘Emergency Hospital Admissions, Prognosis, and Population Mortality in Norway during the First Wave of the Covid-19 Epidemic’. *Scandinavian Journal of Public Health* 50 (6): 795–802. https://doi.org/10.1177/14034948221082959.

Kalanj, Karolina, Ric Marshall, Karl Karol, Mirjana Kujundžić Tiljak, and Stjepan Orešković. 2021. ‘The Impact of COVID-19 on Hospital Admissions in Croatia’. *Frontiers in Public Health* 9 (September): 720948. https://doi.org/10.3389/fpubh.2021.720948.

Karlafti, Eleni, Emmanouil S. Benioudakis, Daniel Paramythiotis, Konstantinos Sapalidis, Georgia Kaiafa, Triantafyllos Didangelos, Antonios Michalopoulos, Isaak Kesisoglou, and Christos Savopoulos. 2021. ‘Does the COVID-19 Pandemic Affect Morbidity and Mortality Rates of Emergency General Surgery? A Retrospective Study from a Single-Center Tertiary Greek Hospital’. *Medicina* 57 (11): 1185. https://doi.org/10.3390/medicina57111185.

Kastritis, Efstathios, Konstantinos Tsitsimpis, Ektoras Anninos, Kimonas Stamatelopoulos, Ioannis Kanakakis, Christos Lampropoulos, Sofia Chatzidou, et al. 2020. ‘Significant Reduction in the Visits to the Emergency Room Department during the COVID-19 Pandemic in a Tertiary Hospital in Greece: Indirect Victims of the Pandemic?’ *Medicine* 99 (52): e23845. https://doi.org/10.1097/MD.0000000000023845.

Kiliç, Pınar, and Halit Nahit Şendur. 2021. ‘Impacts of COVID-19 Pandemic on Computed Tomography Usages in Emergency Department: Cross-Sectional Study’. *Turkiye Klinikleri Journal of Medical Sciences* 41 (3): 274–79. https://doi.org/10.5336/medsci.2021-81381.

Kociejowski, A, C Hobart, R Jina, I Aberman, E Backhurst, A Beaumont, J Crompton, R Sneep, F Cantle, and H Dodhia. 2021. ‘Comparison of Presentations to the Emergency Department during the COVID-19 Pandemic (COPED-C)’. *Journal of Public Health* 43 (4): 731–38. https://doi.org/10.1093/pubmed/fdab059.

Köhler, Franziska, Laura Acar, Anne van den Berg, Sven Flemming, Carolin Kastner, Sophie Müller, Johannes Diers, et al. 2021. ‘Impact of the COVID-19 Pandemic on Appendicitis Treatment in Germany—a Population-Based Analysis’. *Langenbeck’s Archives of Surgery* 406 (2): 377–83. https://doi.org/10.1007/s00423-021-02081-4.

Konson, Alexander, Michael Kuniavsky, Olga Bronshtein, Nethanel Goldschmidt, Shuli Hanhart, Hannah Mahalla, Shir Peri, Shaul Dollberg, and Yaron Niv. 2022. 'Quality of Care Indicator Performance Was Minimally Changed in 2020 despite the COVID-19 Pandemic'. *Israel Journal of Health Policy Research* 11 (1): 9. https://doi.org/10.1186/s13584-022-00516-x.

Kuitunen, Ilari, Ville T. Ponkilainen, Antti P. Launonen, Aleksi Reito, Teemu P. Hevonkorpi, Juha Paloneva, and Ville M. Mattila. 2020. 'The Effect of National Lockdown Due to COVID-19 on Emergency Department Visits'. *Scandinavian Journal of Trauma, Resuscitation and Emergency Medicine* 28 (1): 1–8.

Kurihara, Hayato, Enrico Marrano, Martina Ceolin, Osvaldo Chiara, Roberto Faccincani, Pietro Bisagni, Luca Fattori, et al. 2021. 'Impact of Lockdown on Emergency General Surgery during First 2020 COVID-19 Outbreak'. *European Journal of Trauma and Emergency Surgery* 47 (3): 677–82. https://doi.org/10.1007/s00068-021-01691-3.

Kwok, Edmund, Glenda Clapham, and Samantha Calder-Sprackman. 2021. 'The Impact of COVID-19 Pandemic on Emergency Department Visits at a Canadian Academic Tertiary Care Center'. *Western Journal of Emergency Medicine* 22 (4): 851–59. https://doi.org/10.5811/westjem.2021.2.49626.

Lane, Daniel J., Ian E. Blanchard, Jason E. Buick, Marta Shaw, and Andrew D. McRae. 2021. 'Changes in Presentation, Presenting Severity and Disposition among Patients Accessing Emergency Services during the First Months of the COVID-19 Pandemic in Calgary, Alberta: A Descriptive Study'. *CMAJ Open* 9 (2): E592–601. https://doi.org/10.9778/cmajo.20200313.

Lange, Samantha J., Matthew D. Ritchey, Alyson B. Goodman, Taylor Dias, Evelyn Twentyman, Jennifer Fuld, Laura A. Schieve, et al. 2020. 'Potential Indirect Effects of the COVID-19 Pandemic on Use of Emergency Departments for Acute Life-Threatening Conditions — United States, January–May 2020'. *American Journal of Transplantation* 20 (9): 2612–17. https://doi.org/10.1111/ajt.16239.

Lapsekili, Emin, Mehmet Zeki Buldanlı, and Yąsar Subutay Peker. 2021. 'Comparison of Outcomes of the Patients with Acute Cholecystitis Treated in the COVID-19 Pandemic and Pre-Pandemic Period'. *Revista Da Associação Médica Brasileira* 67 (11): 1681–86. https://doi.org/10.1590/1806-9282.20210727.

Lastrucci, Vieri, Francesca Collini, Silvia Forni, Sara D'Arienzo, Valeria Di Fabrizio, Primo Buscemi, Chiara Lorini, Fabrizio Gemmi, and Guglielmo Bonaccorsi. 2022. 'The Indirect Impact of COVID-19 Pandemic on the Utilization of the Emergency Medical Services during the First Pandemic Wave: A System-Wide Study of Tuscany Region, Italy'. Edited by Francesca Baratta. *PLOS ONE* 17 (7): e0264806. https://doi.org/10.1371/journal.pone.0264806.

Laukkanen, Lauri, Sanna Lahtinen, Janne Liisanantti, Timo Kaakinen, Ari Ehrola, and Lasse Raatiniemi. 2021. 'Early Impact of the COVID-19 Pandemic and Social Restrictions on Ambulance Missions'. *European Journal of Public Health* 31 (5): 1090–95. https://doi.org/10.1093/eurpub/ckab065.

Lawless, Michael, Mark Burgess, and Stephen Bourke. 2022. 'Impact of COVID-19 on Hospital Admissions for COPD Exacerbation: Lessons for Future Care'. *Medicina* 58 (1): 66. https://doi.org/10.3390/medicina58010066.

Lee, Daniel Dongjoo, Wendy Lou, David Rauchwerger, Lucas Chartier, Sameer Masood, and Ahmed Khaled Taher. 2021. 'The Impact of COVID-19 on a Large, Canadian Community Emergency Department'. *Western Journal of Emergency Medicine* 22 (3). https://doi.org/10.5811/westjem.2021.1.50123.

Lee, Stephen, Anthony Santarelli, Heesun Choi, and John Ashurst. 2021. 'Impact of the COVID-19 Pandemic on Emergency Department Transfers to a Higher Level of Care'. *Western Journal of Emergency Medicine* 22 (3). https://doi.org/10.5811/westjem.2021.3.50907.

Lim, Zheng Jie, Mallikarjuna Ponnapa Reddy, Afsana Afroz, Baki Billah, Kiran Shekar, and Ashwin Subramaniam. 2020. ‘Incidence and Outcome of Out-of-Hospital Cardiac Arrests in the COVID-19 Era: A Systematic Review and Meta-Analysis’. *Resuscitation* 157: 248–58.

Loftus, Timothy M., Emily G. Wessling, Daniel S. Cruz, Michael J. Schmidt, Howard S. Kim, Danielle M. McCarthy, and Sanjeev Malik. 2022. ‘Impact of the COVID Pandemic on Emergency Department CT Utilization: Where Do We Go from Here?’ *Emergency Radiology* 29 (5): 879–85. https://doi.org/10.1007/s10140-022-02071-z.

Long, Adrianna, Meghan Fillinger, Michael D April, Ian L Hudson, Wesley Trueblood, Gillian Schmitz, Eric J Chin, Bonnie Hartstein, James A Pfaff, and Steven G Schauer. 2022. ‘Changes in Emergency Department Volumes at the Largest U.S. Military Hospital During the COVID-19 Pandemic’. *Military Medicine* 187 (Special Issue_13): e1538–43. https://doi.org/10.1093/milmed/usab322.

Lopez-Villegas, Antonio, Rafael Jesus Bautista-Mesa, Miguel Angel Baena-Lopez, Antonio Garzon-Miralles, Miguel Angel Castellano-Ortega, Cesar Leal-Costa, and Salvador Peiro. 2022. ‘Impact of the COVID-19 Pandemic on Healthcare Activity in the Regional Hospitals of Andalusia (Spain)’. *Journal of Clinical Medicine* 11 (2): 363. https://doi.org/10.3390/jcm11020363.

Lowe, Jason, Ian Brown, Ram Duriseti, Moises Gallegos, Ryan Ribeira, Elizabeth Pirrotta, and N. Ewen Wang. 2021. ‘Emergency Department Access During COVID-19: Disparities in Utilization by Race/Ethnicity, Insurance, and Income’. *Western Journal of Emergency Medicine* 22 (3). https://doi.org/10.5811/westjem.2021.1.49279.

Lucero, Anthony, Andre Lee, Jenny Hyun, Carol Lee, Chadi Kahwaji, Gregg Miller, Michael Neeki, Joshua Tamayo-Sarver, and Luhong Pan. 2020. ‘Underutilization of the Emergency Department During the COVID-19 Pandemic’. *Western Journal of Emergency Medicine* 21 (6). https://doi.org/10.5811/westjem.2020.8.48632.

Lund, Sarah, Taleen MacArthur, Marianna Martini Fischmann, Justin Maroun, Johnny Dang, James R. Markos, Martin Zielinski, and Daniel Stephens. 2021. ‘Impact of COVID-19 Governmental Restrictions on Emergency General Surgery Operative Volume and Severity’. *The American Surgeon*, April, 000313482110111. https://doi.org/10.1177/00031348211011113.

Ma, Joyce Lok Gee, Vikash Yogaraj, Mustafa Siddiqui, Karanjeet Chauhan, Vicky A. Tobin, and Charles H. C. Pilgrim. 2022. ‘The Impact of COVID-19 on Emergency Cholecystectomy’. *ANZ Journal of Surgery* 92 (3): 409–13. https://doi.org/10.1111/ans.17406.

Maclagan, Laura C., Xuesong Wang, Abby Emdin, Aaron Jones, R. Liisa Jaakkimainen, Michael J. Schull, Nadia Sourial, Isabelle Vedel, Richard H. Swartz, and Susan E. Bronskill. 2022. ‘Visits to the Emergency Department by Community-Dwelling People with Dementia during the First 2 Waves of the COVID-19 Pandemic in Ontario: A Repeated Cross-Sectional Analysis’. *CMAJ Open* 10 (3): E610–21. https://doi.org/10.9778/cmajo.20210301.

Madanelo, Mariana, Carlos Ferreira, Diogo Nunes-Carneiro, André Pinto, Maria Alexandra Rocha, Jorge Correia, Bernardo Teixeira, Gonçalo Mendes, Catarina Tavares, and Sofia Mesquita. 2020. ‘The Impact of the Coronavirus Disease 2019 Pandemic on the Utilisation of Emergency Urological Services’. *BJU International* 126 (2): 256–58.

Masuda, Yoshio, Seth En Teoh, Jun Wei Yeo, Darren Jun Hao Tan, Daryl Lin Jimian, Shir Lynn Lim, Marcus Eng Hock Ong, Audrey L. Blewer, and Andrew Fu Wah Ho. 2022. ‘Variation in Community and Ambulance Care Processes for Out-of-Hospital Cardiac Arrest during the COVID-19 Pandemic: A Systematic Review and Meta-Analysis’. *Scientific Reports* 12 (1): 800. https://doi.org/10.1038/s41598-021-04749-9.

Mathew, Shobi, Nicholas Harrison, Adam D. Chalek, Damon Gorelick, Erin Brennan, Stefanie Wise, Lauren Gandolfo, Brian O'Neil, and Robert Dunne. 2021. 'Effects of the COVID-19 Pandemic on out-of-Hospital Cardiac Arrest Care in Detroit'. *The American Journal of Emergency Medicine* 46 (August): 90–96. https://doi.org/10.1016/j.ajem.2021.03.025.

McGuinness, Matthew J., and Li Hsee. 2020. 'Impact of the COVID-19 National Lockdown on Emergency General Surgery: Auckland City Hospital's Experience.' *ANZ Journal of Surgery* 90 (11): 2254–58. https://doi.org/10.1111/ans.16336.

Meurice, Laure, Pascal Vilain, Laurent Maillard, Philippe Revel, Céline Caserio-Schonemann, and Laurent Filleul. 2021. 'Impact Des Deux Confinements Sur Le Recours Aux Soins d'urgence Lors de l'épidémie de COVID-19 En Nouvelle-Aquitaine (Impact of the Two Lockdowns on the Use of a Hospital Emergency Department during the COVID-19 Epidemic in the Nouvelle-Aquitaine Region)'. *Santé Publique* Vol. 33 (3): 393–97. https://doi.org/10.3917/spub.213.0393.

Mitchell, Rob D, Gerard M O'Reilly, Biswadev Mitra, De Villiers Smit, Jean-Philippe Miller, and Peter A Cameron. 2020. 'Impact of COVID-19 State of Emergency Restrictions on Presentations to Two Victorian Emergency Departments'. *Emergency Medicine Australasia* 32 (6): 1027–33. https://doi.org/10.1111/1742-6723.13606.

Miyagami, Taiju, Yuki Uehara, Taku Harada, Takashi Watari, Taro Shimizu, Ayako Nakamura, Naoya Ogura, Seiko Kushiro, Katsutoshi Masuyama, and Yoshinori Kanai. 2021. 'Delayed Treatment of Bacteremia during the COVID-19 Pandemic'. *Diagnosis* 8 (3): 327–32.

Montero-Pérez, Francisco Javier, and Luís Manuel Jiménez Murillo. 2021. 'Impact of the First COVID-19 Pandemic Wave on the Care and Quality Indicators of a Hospital Emergency Department'. *Emergencias: Revista De La Sociedad Espanola De Medicina De Emergencias* 33 (5): 345–53.

Moon, Jade Y., John B. Miller, Raviv Katz, Thong Ta, Colleen Szypko, Itika Garg, Alice C. Lorch, Matthew F. Gardiner, and Grayson W. Armstrong. 2020. 'The Impact of the COVID-19 Pandemic on Ophthalmic Care at an Eye-Specific Emergency Department in an Outbreak Hotspot'. *Clinical Ophthalmology*, 4155–63.

Motterle, Giovanni, Alessandro Morlacco, Massimo Iafrate, Marta Bianco, Giuliano Federa, Orest Xhafka, Filiberto Zattoni, and Tommaso Prayer-Galetti. 2021. 'The Impact of COVID-19 Pandemic on Urological Emergencies: A Single-Center Experience'. *World Journal of Urology* 39 (6): 1985–89. https://doi.org/10.1007/s00345-020-03264-2.

Müller, Frank, Eva Hummers, Alexandra Jablonka, Tobias Schmidt, and Eva Maria Noack. 2022. 'Auswirkung des COVID-19-Lockdowns auf Rettungseinsätze (Impact of the COVID-19 lockdown on emergency medical service operations)'. *Notfall & Rettungsmedizin* 25 (5): 341–47. https://doi.org/10.1007/s10049-021-00873-1.

Musajee, Mustafa, Lukla Biasi, Narayanan Thulasidasan, Meryl Green, Federica Francia, Martin Arissol, Alpa Lakhani, Stephen Thomas, Sanjay Patel, and Hany Zayed. 2022. 'The Impact of the COVID-19 Pandemic on the Workload, Case Mix and Hospital Resources at a Tertiary Vascular Unit'. *Annals of Vascular Surgery* 80 (March): 104–12. https://doi.org/10.1016/j.avsg.2021.10.021.

Muselli, M., V. Cofini, and L. Mammarella. 2021. 'The Impact of Covid-19 Pandemic on Emergency Services'. *ANNALI DI IGIENE MEDICINA PREVENTIVA E DI COMUNITÀ*. https://doi.org/10.7416/ai.2021.2480.

Nishida, Yasunori, Noriaki Otagiri, and Katsunori Tauchi. 2021. ‘Emergency Abdominal Surgeries Remain Unchanged in the COVID-19 Affected Environment: A Single-center Experience at a Community Hospital in Japan’. *Acute Medicine & Surgery* 8 (1). https://doi.org/10.1002/ams2.623.

Nok, Chun Nok, Sarah Axeen, Sophie Terp, Elizabeth Burner, Daniel Dworkis, Sanjay Arora, and Michael Menchine. 2021. ‘Who Stayed Home Under Safer-at-Home? Impacts of COVID-19 on Volume and Patient-Mix at an Emergency Department’. *Western Journal of Emergency Medicine* 22 (2): 234. https://doi.org/10.5811/westjem.2020.12.49234.

Nopp, Stephan, Karin Janata-Schwatczek, Helmut Prosch, Ihor Shulym, Oliver Königsbrügge, Ingrid Pabinger, and Cihan Ay. 2020. ‘Pulmonary Embolism during the COVID-19 Pandemic: Decline in Diagnostic Procedures and Incidence at a University Hospital’. *Research and Practice in Thrombosis and Haemostasis* 4 (5): 835–41. https://doi.org/10.1002/rth2.12391.

Nourian, Alex, Curran Uppaluri, Michelle Chen, Eric M. Ghiraldi, and Justin I. Friedlander. 2022. ‘Comparison of Management and Outcomes of Symptomatic Urolithiasis During the COVID-19 Pandemic to a Comparative Cohort’. *Urology* 165 (July): 178–83. https://doi.org/10.1016/j.urology.2022.01.019.

Novara, Giacomo, Riccardo Bartoletti, Alessandro Crestani, Cosimo De Nunzio, Jacopo Durante, Andrea Gregori, Giovanni Liguori, Nicola Pavan, Carlo Trombetta, and Alchiede Simonato. 2020. ‘Impact of the COVID-19 Pandemic on Urological Practice in Emergency Departments in Italy’. *BJU International* 126 (2): 245.

O’Brien, Ciara M., Katherine Jung, Wilfred Dang, Hyun-Jung Jang, and Ania Z. Kielar. 2020. ‘Collateral Damage: The Impact of the COVID-19 Pandemic on Acute Abdominal Emergency Presentations’. *Journal of the American College of Radiology* 17 (11): 1443–49. https://doi.org/10.1016/j.jacr.2020.08.010.

O’Connell, R.M., M.A. Khan, M. Amir, M. Bucheeri, W. Khan, I.Z. Khan, and K.M. Barry. 2021. ‘The Impact of COVID-19 on Emergency General Surgery Admissions and Operative Volumes: A Single Centre Experience’. *The Surgeon* 19 (5): e207–12. https://doi.org/10.1016/j.surge.2020.09.013.

Parikh, Keval D., Nikhil H. Ramaiya, Elias G. Kikano, Sree Harsha Tirumani, Jonathan Pierce, Carl Butcher, Jeffrey L. Sunshine, and Donna M. Plecha. 2020. ‘Quantifying the Decrease in Emergency Department Imaging Utilization during the COVID-19 Pandemic at a Multicenter Healthcare System in Ohio’. *Emergency Radiology* 27 (6): 765–72. https://doi.org/10.1007/s10140-020-01848-4.

Porreca, Angelo, Michele Colicchia, Daniele D’Agostino, Michele Amenta, Alfio Corsaro, Stefano Zaramella, Luisa Zegna, et al. 2020. ‘Urology in the Time of Coronavirus: Reduced Access to Urgent and Emergent Urological Care during the Coronavirus Disease 2019 Outbreak in Italy’. *Urologia Internationalis* 104 (7–8): 631–36. https://doi.org/10.1159/000508512.

Poyser, Alicia, Sundeep S. Deol, Lina Osman, Helen J. Kuht, Tharsica Sivagnanasithiyar, Roslyn Manrique, Linda O. Okafor, Ian DeSilva, David Sharpe, and Vijay Savant. 2021. ‘Impact of COVID-19 Pandemic and Lockdown on Eye Emergencies’. *European Journal of Ophthalmology* 31 (6): 2894–2900.

Rajesh, Joel, Gintare Valentelyte, Deborah A. McNamara, and Jan Sorensen. 2022. ‘Impact of the COVID-19 Pandemic on Provision and Outcomes of Emergency Abdominal Surgery in Irish Public Hospitals’. *Irish Journal of Medical Science (1971 -)* 191 (5): 2275–82. https://doi.org/10.1007/s11845-021-02857-z.

Reinke, Caroline E., Huaping Wang, Kyle Thompson, B. Lauren Paton, William Sherrill, Samuel W. Ross, Lynnette Schiffern, and Brent D. Matthews. 2023. 'Impact of COVID-19 on Common Non-Elective General Surgery Diagnoses'. *Surgical Endoscopy* 37 (1): 692–702. https://doi.org/10.1007/s00464-022-09154-5.

Rennert-May, Elissa, Jenine Leal, Nguyen Xuan Thanh, Eddy Lang, Shawn Dowling, Braden Manns, Tracy Wasylak, and Paul E. Ronksley. 2021. 'The Impact of COVID-19 on Hospital Admissions and Emergency Department Visits: A Population-Based Study'. Edited by Sandra C. Buttigieg. *PLOS ONE* 16 (6): e0252441. https://doi.org/10.1371/journal.pone.0252441.

Reschen, Michael E., Jordan Bowen, Alex Novak, Matthew Giles, Sudhir Singh, Daniel Lasserson, and Christopher A. O'Callaghan. 2021. 'Impact of the COVID-19 Pandemic on Emergency Department Attendances and Acute Medical Admissions'. *BMC Emergency Medicine* 21 (1): 143. https://doi.org/10.1186/s12873-021-00529-w.

Rollman, Jeffrey Eric, Robert A. Kloner, Nichole Bosson, James T. Niemann, Marianne Gausche-Hill, Michelle Williams, Christine Clare, et al. 2021. 'Emergency Medical Services Responses to Out-of-Hospital Cardiac Arrest and Suspected ST-Segment–Elevation Myocardial Infarction During the COVID-19 Pandemic in Los Angeles County'. *Journal of the American Heart Association* 10 (12): e019635. https://doi.org/10.1161/JAHA.120.019635.

Rosell Ortiz, Fernando, Patricia Fernández del Valle, Emily C. Knox, Xavier Jiménez Fábrega, José M. Navalpotro Pascual, Inmaculada Mateo Rodríguez, José I. Ruiz Azpiazu, et al. 2020. 'Influence of the Covid-19 Pandemic on out-of-Hospital Cardiac Arrest. A Spanish Nationwide Prospective Cohort Study'. *Resuscitation* 157 (December): 230–40. https://doi.org/10.1016/j.resuscitation.2020.09.037.

Rudnicki, Yaron, Hagai Soback, Ori Mekiten, Guy Lifshiz, and Shmuel Avital. 2022. 'The Impact of COVID-19 Pandemic Lockdown on the Incidence and Outcome of Complicated Appendicitis'. *Surgical Endoscopy* 36 (5): 3460–66. https://doi.org/10.1007/s00464-021-08667-9.

Russo, Vincenzo, Giulio Boggian, Maria Giulia Bolognesi, Domenico Maria Carretta, Simone Cencetti, Domenica De Laura, Enzo Hrovatin, et al. 2021. 'The Impact of COVID-19 Outbreak on Syncope Units Activities in Italy: A Report from the Italian Multidisciplinary Working Group on Syncope (GIMSI)'. *International Journal of Environmental Research and Public Health* 18 (17): 9194. https://doi.org/10.3390/ijerph18179194.

Sagy, Yael Wolff, Assi Cicurel, Erez Battat, Walid Saliba, and Gil Lavie. 2022. 'The Impact of COVID-19 Pandemic on Emergency Department Visits and Associated Mortality during 14 Months of the Pandemic in Israel'. *Internal and Emergency Medicine* 17 (6): 1699–1710. https://doi.org/10.1007/s11739-022-02991-1.

Şan, İshak, Eren Usul, Burak Bekgöz, and Semih Korkut. 2021. 'Effects of COVID-19 Pandemic on Emergency Medical Services'. *International Journal of Clinical Practice* 75 (5). https://doi.org/10.1111/ijcp.13885.

Sarc, Irena, Alesa Lotric Dolinar, Tina Morgan, Joze Sambt, Kristina Ziherl, Dalibor Gavric, Julij Selb, Ales Rozman, and Petra Dosenovic Bonca. 2022. 'Mortality, Seasonal Variation, and Susceptibility to Acute Exacerbation of COPD in the Pandemic Year: A Nationwide Population Study'. *Therapeutic Advances in Respiratory Disease* 16 (January): 175346662210810. https://doi.org/10.1177/17534666221081047.

Scquizzato, Tommaso, Giovanni Landoni, Andrea Paoli, Rosalba Lembo, Evgeny Fominskiy, Artem Kuzovlev, Valery Likhvantsev, and Alberto Zangrillo. 2020. 'Effects of COVID-19 Pandemic on out-of-Hospital Cardiac Arrests: A Systematic Review'. *Resuscitation* 157 (December): 241–47. https://doi.org/10.1016/j.resuscitation.2020.10.020.

Sekine, Ichiro, Haruki Uojima, Hiroshi Koyama, Tadashi Kamio, Morihiko Sato, Tadatsugu Yamamoto, Kiyomitsu Fukaguchi, Hiroyuki Fukui, and Hiroshi Yamagami. 2020. 'Impact of Non-pharmaceutical Interventions for the COVID-19 Pandemic on Emergency Department Patient Trends in Japan: A Retrospective Analysis'. *Acute Medicine & Surgery* 7 (1). https://doi.org/10.1002/ams2.603.

Sharperson, Camara, Tarek N. Hanna, Keith D. Herr, Matthew E. Zygmont, Roger L. Gerard, and Jamlik-Omari Johnson. 2021. 'The Effect of COVID-19 on Emergency Department Imaging: What Can We Learn?' *Emergency Radiology* 28: 339–47.

Shreffler, Jacob, Hugh Shoff, J. Jeremy Thomas, and Martin Huecker. 2021. 'Brief Report: The Impact of COVID-19 on Emergency Department Overdose Diagnoses and County Overdose Deaths'. *The American Journal on Addictions* 30 (4): 330–33. https://doi.org/10.1111/ajad.13148.

Siegel, Robert, Sven Hohenstein, Stefan Anders, Martin Strik, Ralf Kuhlen, and Andreas Bollmann. 2022. 'Access to Surgery and Quality of Care for Acute Cholecystitis During the COVID-19 Pandemic in 2020 and 2021 — an Analysis of 12,545 Patients from a German-Wide Hospital Network'. *Journal of Gastrointestinal Surgery* 26 (7): 1462–71. https://doi.org/10.1007/s11605-022-05318-9.

Sless, Ryan Taylor, Nathaniel Edward Hayward, Paul MacDaragh Ryan, Conor Deasy, and Kantikiran Dasari. 2021. 'Emergency Department Attendances during the COVID-19 Pandemic: A Retrospective Analysis of Attendances Following Irish Governmental Pandemic Measures'. *Emergency Medicine Journal* 38 (6): 439–45. https://doi.org/10.1136/emermed-2020-209992.

Soares, William E., Edward R. Melnick, Bidisha Nath, Gail D'Onofrio, Hyung Paek, Rachel M. Skains, Lauren A. Walter, et al. 2022. 'Emergency Department Visits for Nonfatal Opioid Overdose During the COVID-19 Pandemic Across Six US Health Care Systems'. *Annals of Emergency Medicine* 79 (2): 158–67. https://doi.org/10.1016/j.annemergmed.2021.03.013.

Stöwhas, Mathias, and Hans Lippert. 2021. 'Impact of COVID-19 outbreak on emergency department admissions in a specialized hospital (Entwicklung der Notaufnahmefallzahlen eines Schwerpunktversorgers im Verlauf der Corona-Pandemie in Mecklenburg-Vorpommern)'. *Gesundheitswesen (Bundesverband der Arzte des Offentlichen Gesundheitsdienstes (Germany))* 83 (04): 265–73. https://doi.org/10.1055/a-1384-0508.

Vallès, Katherine F, Miriam Y Neufeld, Elisa Caron, Sabrina E Sanchez, and Tejal S Brahmbhatt. 2021. 'COVID-19 Pandemic and the Cholecystitis Experience at a Major Urban Safety-Net Hospital'. *Journal of Surgical Research* 264 (August): 117–23. https://doi.org/10.1016/j.jss.2021.02.037.

Venkatesh, Arjun K., Alexander T. Janke, Li Shu-Xia, Craig Rothenberg, Pawan Goyal, Aisha Terry, and Michelle Lin. 2021. 'Emergency Department Utilization for Emergency Conditions During COVID-19'. *Annals of Emergency Medicine* 78 (1): 84–91. https://doi.org/10.1016/j.annemergmed.2021.01.011.

Vest, Agathe, Chafik Keilani, Philippe Chaumet-Riffaud, Pierre-Olivier Barale, Eric Tuil, Sarah Ayello-Scheer, Edouard Koch, et al. 2022. 'Incidence and Characteristics of Rhegmatogenous Retinal Detachment during Coronavirus-19 Pandemic: A French Study'. *European Journal of Ophthalmology* 32 (6): 3644–49. https://doi.org/10.1177/11206721221080810.

Vissio, Elena, Enrico Costantino Falco, Gitana Scozzari, Antonio Scarmozzino, Do An Andrea Trinh, Mario Morino, Mauro Papotti, Luca Bertero, and Paola Cassoni. 2021. 'The Adverse Impact of the COVID-19 Pandemic on Abdominal Emergencies: A Retrospective Clinico-Pathological Analysis'. *Journal of Clinical Medicine* 10 (22): 5254. https://doi.org/10.3390/jcm10225254.

Vollmer, Michaela A. C., Sreejith Radhakrishnan, Mara D. Kont, Seth Flaxman, Samir Bhatt, Ceire Costelloe, Kate Honeyford, et al. 2021. 'The Impact of the COVID-19 Pandemic on Patterns of Attendance at Emergency Departments in Two Large London Hospitals: An Observational Study'. *BMC Health Services Research* 21 (1): 1008. https://doi.org/10.1186/s12913-021-07008-9.

Walker, Laura E., Heather A. Heaton, Ryan J. Monroe, R. Ross Reichard, Monica Kendall, Aidan F. Mullan, and Deepi G. Goyal. 2020. 'Impact of the SARS-CoV-2 Pandemic on Emergency Department Presentations in an Integrated Health System'. *Mayo Clinic Proceedings* 95 (11): 2395–2407. https://doi.org/10.1016/j.mayocp.2020.09.019.

Wartelle, Adrien, Farah Mourad-Chehade, Farouk Yalaoui, Jan Chrusciel, David Laplanche, and Stéphane Sanchez. 2021. 'Effect of the COVID-19 Pandemic Lockdown on Non-COVID-19 Emergency Department Visits in Eastern France: Reduced Risk or Avoidance Behavior?' *Public Health in Practice* 2 (November): 100109. https://doi.org/10.1016/j.puhip.2021.100109.

Westgard, Bjorn C., Matthew W. Morgan, Gabriela Vazquez-Benitez, Lauren O. Erickson, and Michael D. Zwank. 2020. 'An Analysis of Changes in Emergency Department Visits After a State Declaration During the Time of COVID-19'. *Annals of Emergency Medicine* 76 (5): 595–601. https://doi.org/10.1016/j.annemergmed.2020.06.019.

Wyatt, Steven, Mohammed A Mohammed, Elizabeth Fisher, Ruth McConkey, and Peter Spilsbury. 2021. 'Impact of the SARS-CoV-2 Pandemic and Associated Lockdown Measures on Attendances at Emergency Departments in English Hospitals: A Retrospective Database Study'. *The Lancet Regional Health - Europe* 2 (March): 100034. https://doi.org/10.1016/j.lanepe.2021.100034.

Zeppieri, Marco, Maria Letizia Salvetat, and Carlo Salati Salati. 2021. 'The Influence of COVID-19 Lockdowns on Presentation with Spontaneous Posterior Vitreous Detachment to the Emergency Department in Italy'. *Frontiers in Bioscience-Elite* 13 (2): 259. https://doi.org/10.52586/E883.
