# Supplementary material for: Acute care pathway assessed through performance indicators during the COVID-19 pandemic in OECD countries (2020–2021): a scoping review
Source: BMC Emerg Med. 2024 Jan 26;24:19. doi: 10.1186/s12873-024-00938-7 (PMC10811879; doi:10.1186/s12873-024-00938-7)

Additional file 5 – Analysis of specific indicator trends by country, clinical entity, and/or diagnostic procedures (.pdf)

Indicators related to general emergent and urgent care: 1- Pre-hospital services

Indicators' Trends (n) by country n=225

**Decrease**	**110**
Australia	42
Canada	28
Finland	13
Turkey	11
US	8
Italy	3
Japan	3
Germany	2
**Increase**	**48**
US	13
Australia	11
Finland	9
Canada	7
Turkey	4
Japan	3
Belgium	1
**Stable**	**67**
Australia	20
Canada	20
Japan	17
Germany	9
Finland	1

Volume of patients contacting Emergency Medical Services (n=225 indicators)

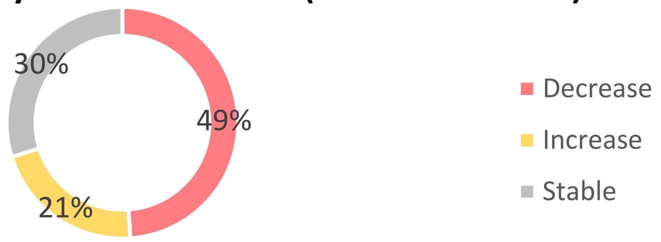

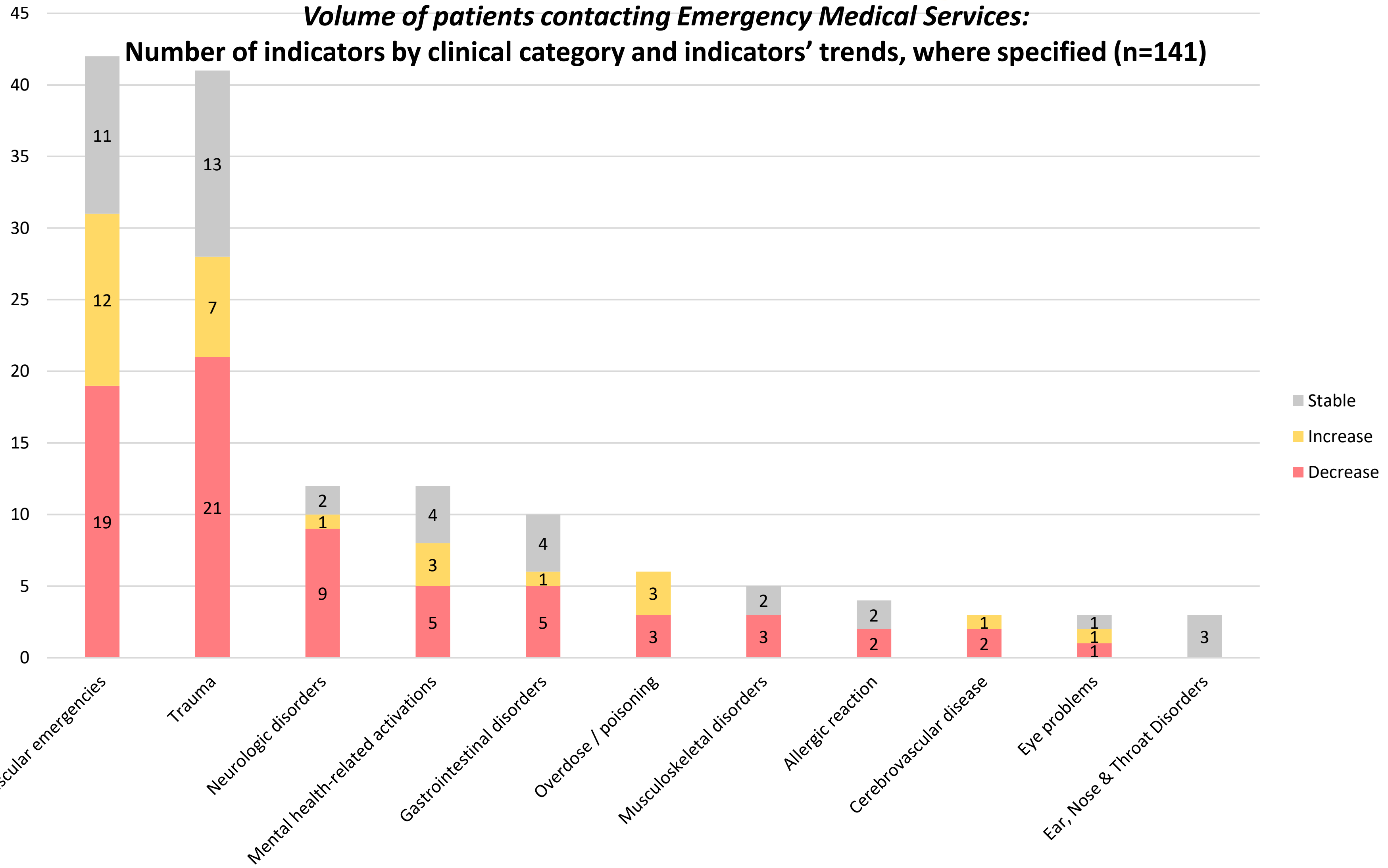

Indicators related to general emergent and urgent care: 1 - Pre-hospital services

Survival rate in patients with out-of-hospital cardiac arrest

Number of indicators and trends by country

**Decrease**	**61**
Spain	37
US	16
Australia	2
Canada	2
Finland	2
Germany	1
Belgium	1
**Increase**	**7**
Spain	4
US	1
Belgium	1
Canada	1
**Stable**	**13**
Germany	5
US	3
Spain	3
Belgium	1
Korea	1
**Total**	**81**

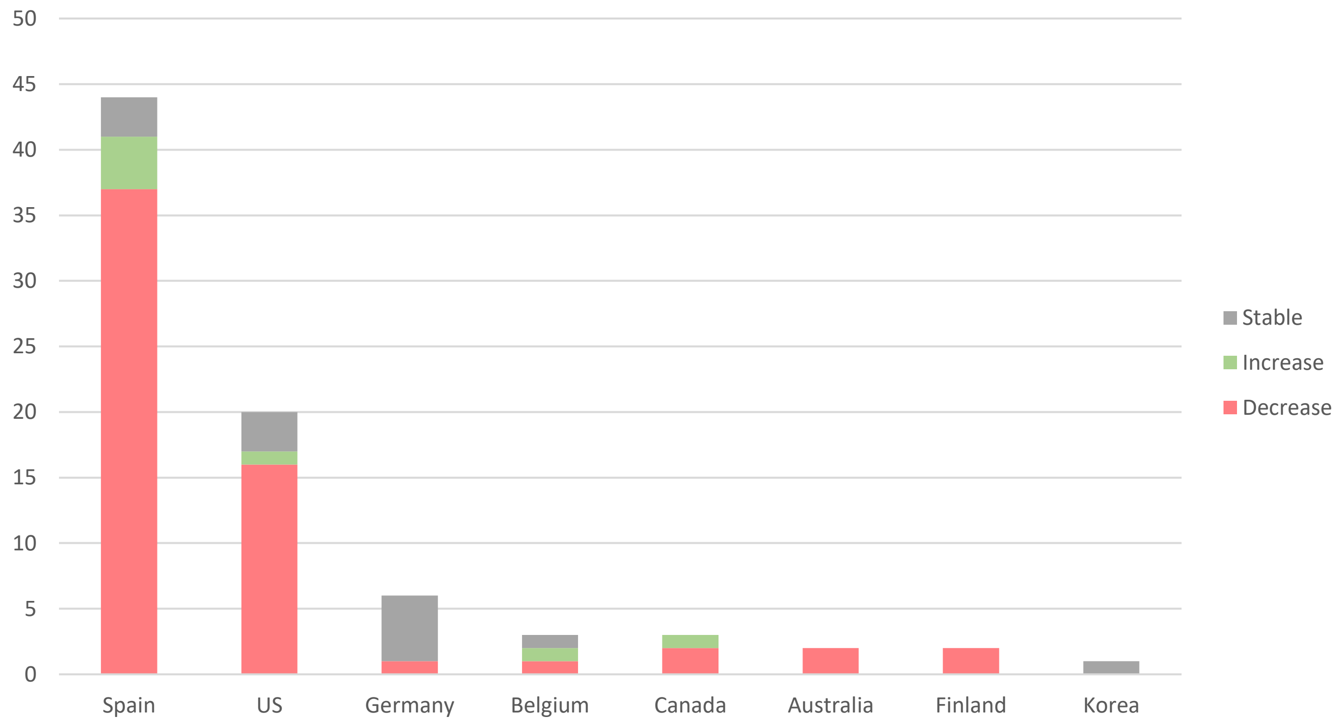

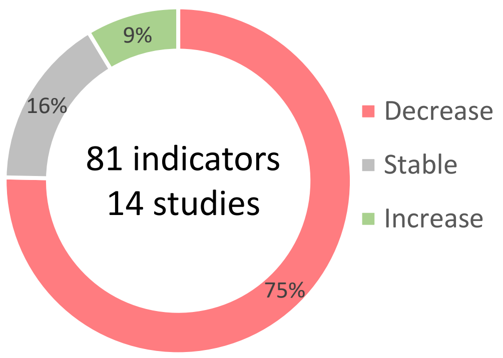

Indicators related to general emergent and urgent care: 2 - Admission to the Emergency Department

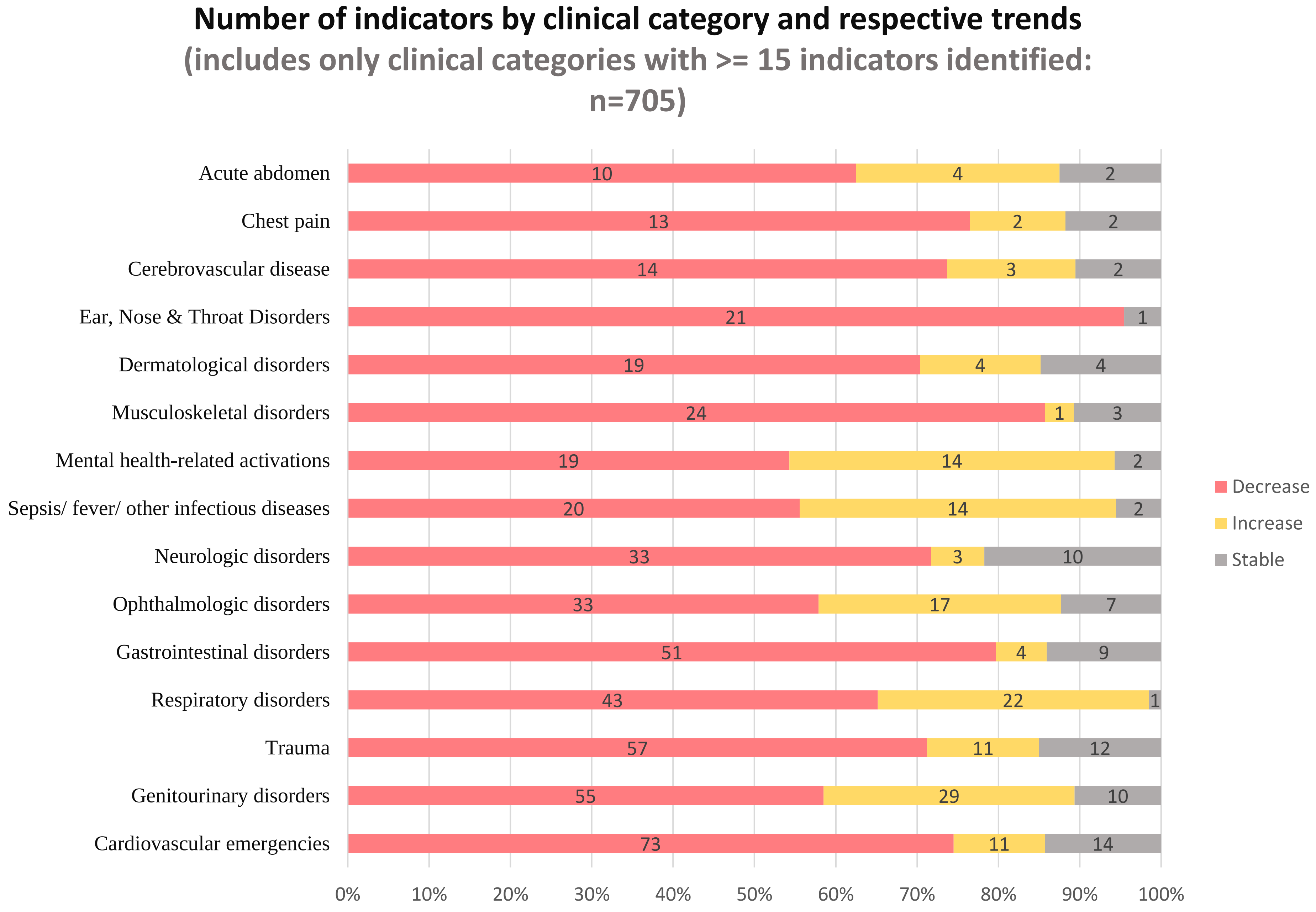

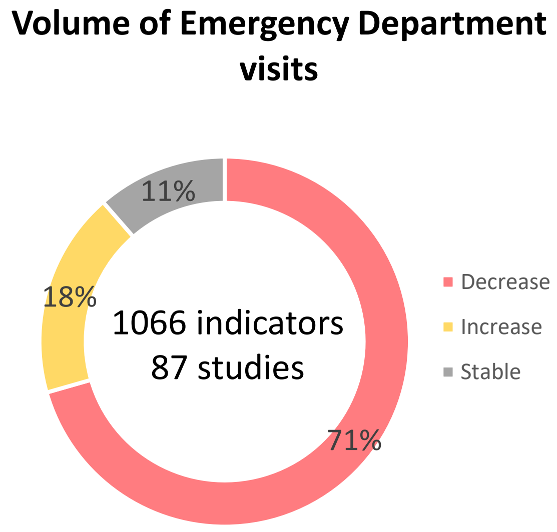

Indicators related to general emergent and urgent care: 3 - Diagnosis

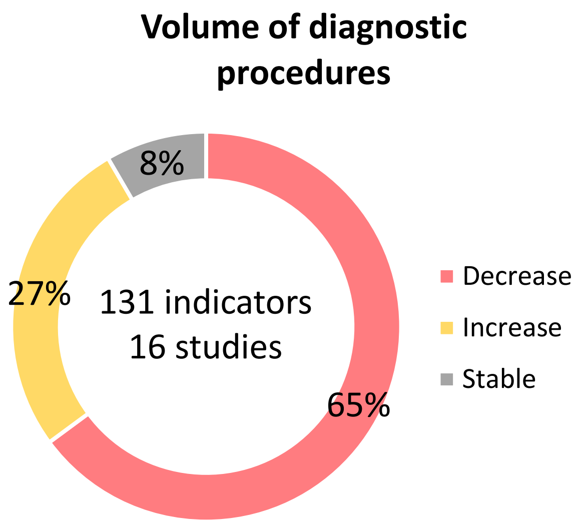

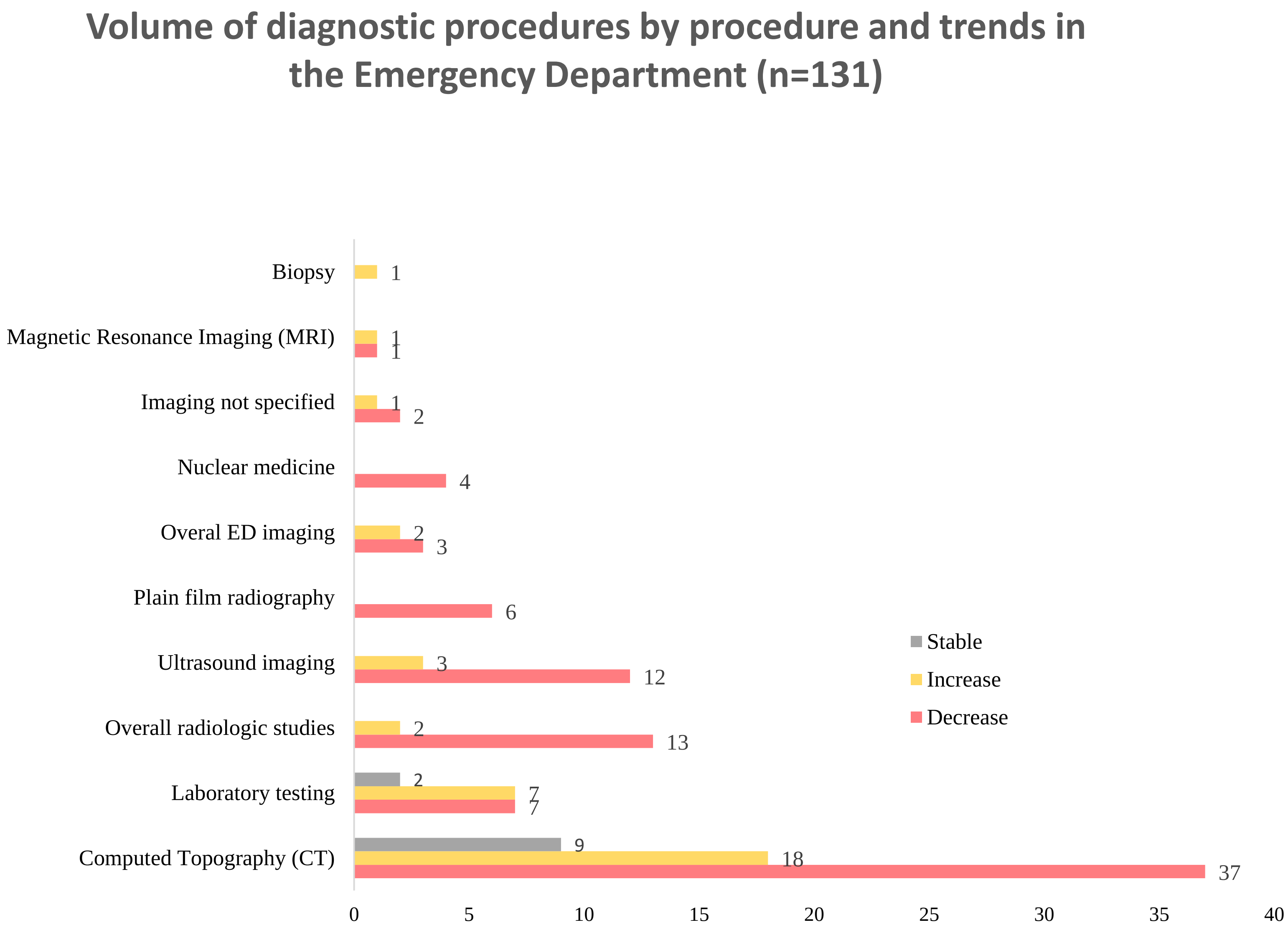

	Trends			
**Diagnostic procedure**	**Decrease**	**Increase**	**Stable**	**Total**
Computed Topography (CT)	37	18	9	64
Laboratory testing	7	7	2	16
Overall radiologic studies	13	2		15
Ultrasound imaging	12	3		15
Plain film radiography	6			6
Overal ED imaging	3	2		5
Nuclear medicine	4			4
Imaging not specified	2	1		3
Magnetic Resonance Imaging (MRI)	1	1		2
Biopsy		1		1
**Total**	**85**	**35**	**11**	**131**

Indicators related to general emergent and urgent care: 4 - Treatment

Number of indicators by trend per country (n=106)

**Decrease**	**54**
US	38
Italy	7
UK	4
Germany	1
Turkey	1
France	1
Ireland	1
Switzerland	1
**Increase**	**31**
US	18
Italy	9
Turkey	2
Ireland	1
Portugal	1
**Stable**	**21**
US	13
Italy	3
UK	2
Switzerland	2
Turkey	1

Volume of therapeutic interventions

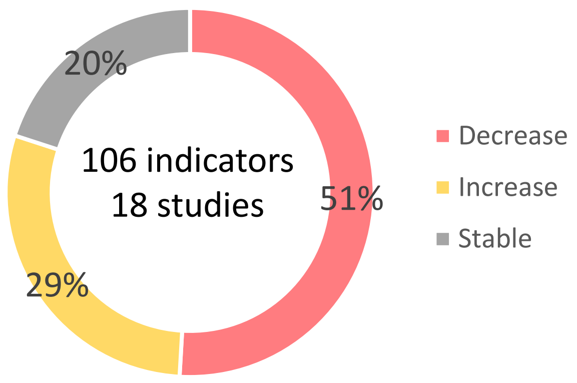

Volume of therapeutic invasive procedures (n=45; 5 studies)

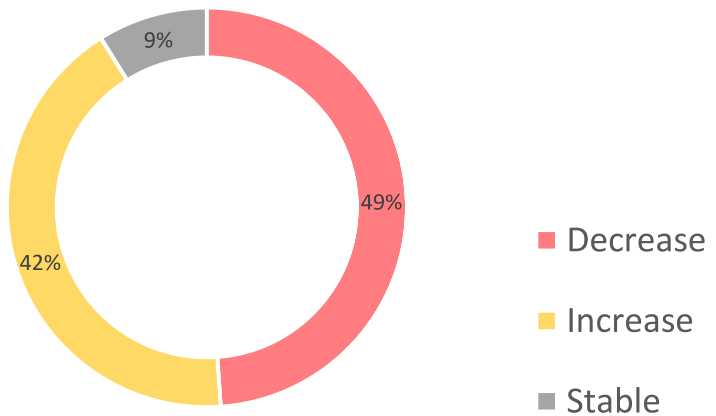

Volume of urgent surgical interventions (n=58)

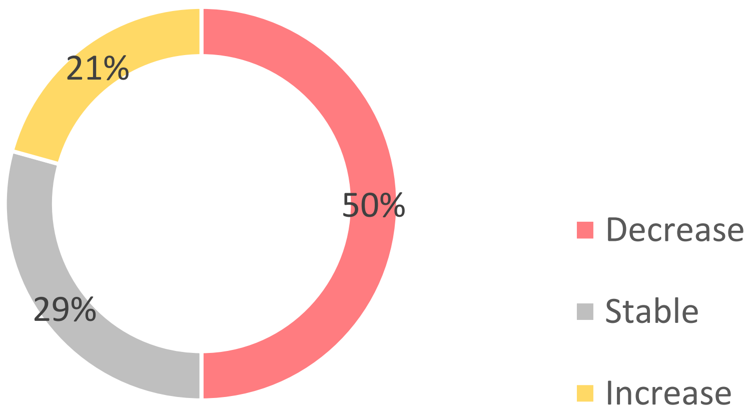

Supplement: Supplementary file 5 — Additional file 5. Analysis of specific indicator trends by country, clinical entity, and/or diagnostic procedures. [file 12873_2024_938_MOESM5_ESM.pdf]
